# A systems biology approach to the global analysis of transcription factors in colorectal cancer

**DOI:** 10.1186/1471-2407-12-331

**Published:** 2012-08-01

**Authors:** Meeta P Pradhan, Nagendra KA Prasad, Mathew J Palakal

**Affiliations:** 1 School of Informatics, Indiana University Purdue University Indianapolis, Indianapolis, IN 46202, USA; 2 Indiana University Melvin and Bren Simon Cancer Center, Indiana University Purdue University Indianapolis, Indianapolis, IN, 46202, USA

## Abstract

**Background:**

Biological entities do not perform in isolation, and often, it is the nature and degree of interactions among numerous biological entities which ultimately determines any final outcome. Hence, experimental data on any single biological entity can be of limited value when considered only in isolation. To address this, we propose that augmenting individual entity data with the literature will not only better define the entity’s own significance but also uncover relationships with novel biological entities.

To test this notion, we developed a comprehensive text mining and computational methodology that focused on discovering new targets of one class of molecular entities, transcription factors (TF), within one particular disease, colorectal cancer (CRC).

**Methods:**

We used 39 molecular entities known to be associated with CRC along with six colorectal cancer terms as the *bait list*, or list of search terms, for mining the biomedical literature to identify CRC-specific genes and proteins. Using the literature-mined data, we constructed a global TF interaction network for CRC. We then developed a multi-level, multi-parametric methodology to identify TFs to CRC.

**Results:**

The small bait list, when augmented with literature-mined data, identified a large number of biological entities associated with CRC. The relative importance of these TF and their associated modules was identified using functional and topological features. Additional validation of these highly-ranked TF using the literature strengthened our findings. Some of the novel TF that we identified were: SLUG, RUNX1, IRF1, HIF1A, ATF-2, ABL1, ELK-1 and GATA-1. Some of these TFs are associated with functional modules in known pathways of CRC, including the Beta-catenin/development, immune response, transcription, and DNA damage pathways.

**Conclusions:**

Our methodology of using text mining data and a multi-level, multi-parameter scoring technique was able to identify both known and novel TF that have roles in CRC. Starting with just one TF (SMAD3) in the bait list, the literature mining process identified an additional 116 CRC-associated TFs. Our network-based analysis showed that these TFs all belonged to any of 13 major functional groups that are known to play important roles in CRC. Among these identified TFs, we obtained a novel six-node module consisting of ATF2-P53-JNK1-ELK1-EPHB2-HIF1A, from which the novel JNK1-ELK1 association could potentially be a significant marker for CRC.

## Background

Advances in the field of bioinformatics have improved the ability to glean useful information from high-density datasets generated from advanced, technology-driven biomedical investigations. However, deriving actionable, hypothesis-building information by combining data from experimental, mechanistic, and correlative investigations with gene expression and interaction data still presents a daunting challenge due to the diversity of the available information, both in terms of their type and interpretation. Because of this, there is a clear need for custom-designed approaches that fit the biology or disease of interest.

Gene expression datasets have been widely used to identify genes and pathways as markers for the specific disease or outcome to which they are linked [1-4]. However, gene expression datasets used alone cannot identify relationships between genes within the system of interest; identification of these relationships also requires integration of interaction networks so that changes in gene expression profiles can be fully understood. One process in which this problem has become particularly important is that of gene prioritization, or the identification of potential marker genes for a specific disease from a pool of disease-related genes. Earlier studies on associating genes with disease were done using linkage analysis [5]. Many computational approaches using functional annotation, gene expression data, sequence based knowledge, phenotype similarity have since been developed to prioritize genes, and recent studies have demonstrated the application of system biology approaches to study the disease relevant gene prioritization.

For example, five different protein-protein interaction networks were analysed using sequence features and distance measures to identify important genes associated with specific hereditary disorders [6]. In other studies, chromosome locations, protein-protein interactions, gene expression data, and loci distance were used to identify and rank candidate genes within disease networks [6-9]. The “guilt by association” concept has also been used to discover disease-related genes by identifying prioritized genes based on their associations [7,10]. Network properties [11,12] have also been used to correlate disease genes both with and without accompanying expression data [11].

Integration of more heterogeneous data has also been utilized in identification of novel disease-associated genes. Examples of such integration include CIPHER, a bioinformatics tool that uses human protein-protein interactions, disease-phenotypes, and gene-phenotypes to order genes in a given disease [13]; use of phenome similarity, protein-protein interactions, and knowledge of associations to identify disease-relevant genes [14]; and machine-learning methods and statistical methods utilizing expression data used to rank the genes in a given differential-expression disease network [15-18] and in 1500 Mendelian disorders [19]. Utilization of literature mining, protein-protein interactions, centrality measures and clustering techniques were used to predict disease-gene association (prostate, cardiovascular) [20-23], while integration of text-mining with knowledge from various databases and application of machine-learning-based clustering algorithms was used to understand relevant genes associated with breast cancer and related terms [24]. In addition to CIPHER, additional bioinformatics tools include Endeavour, which ranks genes based on disease/biological pathway knowledge, expression data, and genomic knowledge from various datasets [25], and BioGRAPH, which explains a concept or disease by integrating heterogeneous data [26]. Most of these described methods, while using a variety of approaches, still use the Human Protein Reference Database (HPRD, http://www.hprd.org) as the knowledge base for protein-protein interactions. The variation in these approaches to achieving comparable goals demonstrates that using a single feature cannot ease the complexity associated with finding disease-gene, disease-phenotype, and gene-phenotype associations. Moreover, the need for integration of the described features is more pertinent for complex diseases, such as cancer. To the best of our knowledge, this integrated approach has not been studied in terms of transcription factor (TF) interaction networks in colorectal cancer (CRC).

It is well-established that TFs are the master regulators of embryonic development, as well as adult homeostasis, and that they are regulated by cell signalling pathways via transient protein interactions and modifications [27,28]. A major challenge faced by biologists is the identification of the important TFs involved in any given system. Though advances in genomic sequencing provided many opportunities for deciphering the link between the genetic code and its biological outcome, the derivation of meaningful information from such large datasets is, as stated earlier, still challenging. The difficulty is largely due to the manner in which TFs function since TFs interacts with multiple regulatory regions of other TFs, ancillary factors, and chromatin regulators in a reversible and dynamic manner to elicit a specific cellular response [29]. While the specific focus on TFs within CRC for this paper is due to their significant regulatory roles, the focus on CRC is four-fold. First, this effort is part of a major, collaborative multi-institute initiative on CRC in the state of Indiana called cancer care engineering (CCE) that involves the gathering of a large body of –omics data from thousands of healthy individuals and patients for the purpose of development of approaches for preventive, diagnostic, and therapeutic clinical applications of this data. Second, in spite of major breakthroughs in understanding the molecular basis of CRC, it continues to present a challenging problem in cancer medicine. CRC has one of the worst outcomes of most known cancers, with significantly lower survival rates than those of uterine, breast, skin, and prostate cancers. Early detection of CRC requires invasive procedures due to the fact that knowledge of useful biomarkers in CRC is relatively lacking and that the drugs currently approved for treatment of CRC are cytotoxic agents that aim to specifically treat advanced disease. Currently, most patients with early stage CRC are not offered adjuvant therapies, as these are associated with significant toxicities and marginal benefits. It is necessary to identify targeted therapeutics for both early CRC, to decrease the toxicity and enable adjuvant therapies to prevent disease progression, and later-stage CRC, to prevent mortality. Third, even though TFs play a major role in CRC, still there is no global TF interaction network analysis reported for this disease. Tying in with the need for a global TF interaction network analysis in CRC, the focus on CRC is lastly due to the need for identification of CRC-specific TFs as potential disease markers, and here we demonstrate the ability of a bioinformatics approach incorporating knowledge from the literature, topological network properties, and biological features to achieve this goal.

Our goal in this study was thus to obtain a TF interaction network for CRC utilizing a bibliomics approach – i.e., by extracting knowledge from PubMED abstracts and ranking TFs according to their topological and biological importance in the network. As explained earlier, understanding of a disease-gene association necessitates multiple features, which our methodology incorporated by augmenting a set of experimental data with relevant literature data to extract and correlate TFs that have so far not been found to be associated with CRC. We have demonstrated that using literature-generated, domain-specific knowledge combined with network and biological properties will yield a CRC-specific TF interaction network that is biologically significant. The TFs identified by this approach represent a pool of potentially novel drug targets and/or biomarkers, which can be narrowed down to a rank-ordered list for further analysis by domain experts for further experimental validations. While this is the first report identifying a TF interaction network for CRC using such an approach, our methodology is broadly applicable, simple, and efficient, especially for preliminary stages of investigation.

## Methods

### Overview of the text-mining strategy

**Figure 1:**
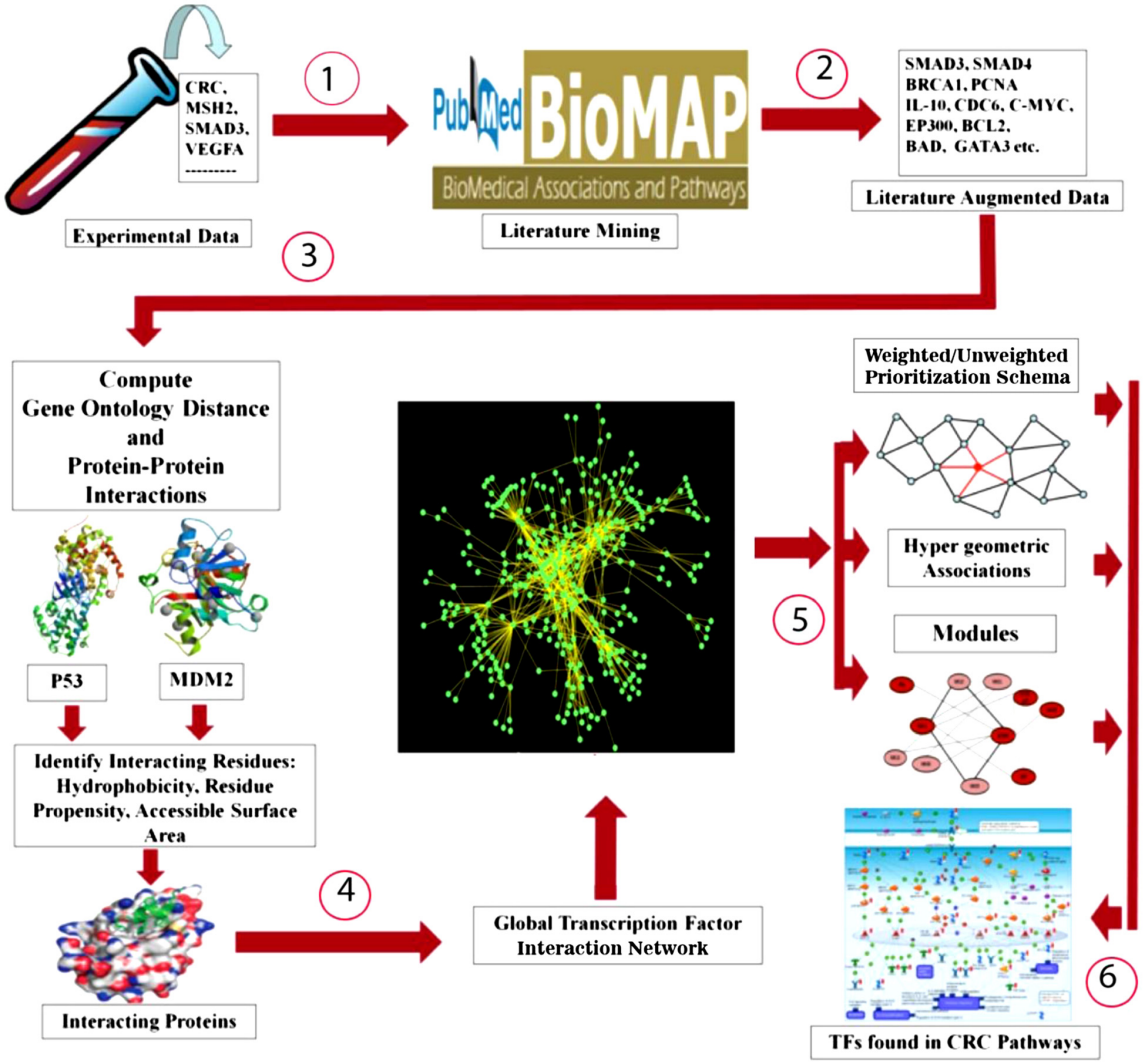
**Methodology for identifying global transcription factor-interactome and important transcription factors in CRC.** Depicts the overall methodology used to prioritize the TFs: (1) Data collection from peer reviews; (2) Discovery of associations using BioMAP (literature augmented data); (3) Validation of BioMAP associations using Gene Ontology distance and protein-protein interactions; (4) Construction of the global TF interaction network; (5) Ranking of TFs using multi-level, multi-parametric using: (i) weighted/un-weighted prioritization schema, (ii) hypergeometric associations, and (iii) Modules; and (6) Validation of TFs by pathway analysis.

Our strategy involved six major steps as shown in Figure 1:

1 Collection and pre-processing of data

2 Discovery of associations using BioMAP (Literature Augmented Data)

3 Validation of BioMAP associations using Gene Ontology Distance and Protein-Protein Interactions

4 Construction of TF interaction network (termed a global interaction network since all available PubMed literature was considered)

(a) Annotation of nodes using topological parameters

5 Ranking of TFs using multi-level, multi-parametric features

(a) Un-weighted/weighted node prioritization

(b) Hyper geometric associations

(c) Construction of functional module

6 Validation of TFs (found in CRC pathways)via pathway analysis

Each of these steps is described below in detail:

### Data collection and pre-processing

Previous work in CRC has identified various disease-relevant anomalies in genes, including *hMLH1* and *MSH2*[3,30,31], *MLH3* with *hMLH1*[31], *NEDD41* along with *PTEN* mutation [32,33], Axin in association with Wnt signalling pathways [34], *MUC2*/*MUC1*[35] and co-expression of *IGFIR*, *EGFR* and *HER2*[36,37], and *p53* and *APC* mutations [37]. Several specific TFs, in addition to playing roles in DNA repair and cell signalling defects, are known to play major roles in CRC. For example *STAT3*, *NF-kB*, and *c-Jun* are oncogenic in CRC [38]. *HOXO9*, *p53*, *c-Myc,* and *β-catenin* together with *Tcf*/*Lef* and *MUC1*[39] and *SOX4*, as well as high levels of the *CBFB* and *SMARCC1* TFs have all been associated with CRC [40]. Using these experimental studies reported in the literature, we manually collected 45 keywords that are well understood and validated in relation to CRC. This initial list, called the ‘bait list’, is given in Table 1. The 39 biological entities in this list were manually evaluated using the criteria that each entity must have a minimum of three references reported in the literature; notably, the bait list contained only one TF, SMAD3. The remaining six terms were related to CRC terminology/types (e.g., colon rectal cancer, colorectal cancer, and CRC). This list was used with BioMAP, a literature mining tool developed and designed in-house to find associations among biological entities such as genes, proteins, diseases, and pathways [41], to retrieve and carry out literature mining on abstracts from PubMed.

**Table 1:** Keywords used for literature mining

**Gene/pathway**	**Association with CRC**	**Ref**
*hMLH1/*DNA repair	Genetic or epigenetic inactivation	[3,98]
*MSH2*/DNA repair	Genetic or epigenetic inactivation	[2]
*MLH3*/DNA repair	Dominant negative mutations inhibit *hMLH1* function	[30,31]
*MYH*/Development	Attenuate CRC in association with *FAP*	[4,99]
*CDK8*/cell cycle regulation	*CDK8* Inhibition activates Wnt/b-catenin pathway	[100,101]
*DCC*	Genetic loss	[102]
*IGF-IR/IGF-IR*, *EGFR* and *HER2* receptor tyrosine kinase signalling	Co-expression in advanced stages	[36]
*TGFBR1*/*TGF*-beta signalling pathway	Inhibits/prevents CRC	[103,104]
*Axin2*/Cytoskeleton remodelling	Mutations activates *Wnt* signalling	[34]
*APC*/Cell cycle	Genetic loss	[105,106]
*b-Raf*/*Ras* signalling pathway	Mutations are prognostic	[107,108]
*MSH6*/DNA damage	Mutations in *HNPCC*	[109,110]
*PTEN*/cell signalling	Genetic loss or functional inactivation linked to poor survival	[32,33]
*CXCL12* and *CXCR4*/Immune response – signalling pathway	Inverse relationship between *CXCL12* and *CXCR4*, with over-expression of *CXCL12* and down-regulation of *CXCR4* are linked to tumor progression	[111]
*RAD18*/DNA damage	Polymorphism at *Arg302Gln*	[112,113]
*c-Met*/*HGF* signalling pathway	Over-expression linked to tumor progression	[114]
*HG*/*HGF* signalling pathway	Over-expression *HGF* in association with c-Met linked to metastasis	[115]
*MACC1*/signalling pathway	Over-expression associated with metastasis	[116]
*CASPASE-3*/apoptosis-*FAS* signalling/*TNFR1*/*caspase-cascade*		[117][118]
*CASP10*/*caspase-cascade*	Somatic mutations linked to pathogenesis	[119]
*NAT1*/metabolic pathways	Genetic mutations	[120,121]
*GSTM1*/detoxification pathway	*GSTM1* expression associated with tumor progression	[122]
*GSTT1*/cell cycle	*GSTT1* expression associated with high risk of CRC	[122]
*CYP2C9*/lipid metabolism	High risk associated with CYP2C9*1 gene	[123], [124]
*Bcl-2*/Apoptosis-*FAS* signalling/*TNFR1* signalling	Loss of expression associated with stage II relapse	[125]
*PRMT1*/DNA repair	Expression of gene variant associated with CRC	[126,127]
*SMAD3*/Cytoskeleton remodelling	Expression is associated with the survival rate of CRC	[128]
*IGFBP1*/*IGF Beta receptor* signalling pathway	Expression is inversely proportional to survival rate in CRC	[129]
*PDGFBB*/*PDGF* signalling pathway	Higher expression associated with low survival rate	[130]
*PDGFRB*/*PDGF* signalling pathway	Higher expression associated with CRC tumor stroma	[131]
*PLK1*/cell cycle	Higher expression and a prognostic factor in CRC	[132]
*IFITM1*/*Beta-catenin* signalling pathway	Expression identified in CRC, important for pathogenesis, metastasis and potential biomarker	[133]
*MBL2*/*lectin* pathway	Very population specific. Two school of thought (yes/no)	NCI bulletin-April-17,2007
*PMS2*/DNA repair	Loss in expression associated with CRC	[134]
*CXCL2*/Apoptotic pathways	Elevated expression associated with CRC	[135]
*IGF1R*/*IGFR* signalling pathway	Regulates the expression of *VEGF* expression. Can be used as prognostic factor.	[136]
*CYP27B1*/*Vitamin D* pathway	Enzyme identified to be associated with CRC- but more studies need to be performed	[137]
*CYP24*/*Vitamin D* pathway	Useful gene/SNP/precursor for chemotherapy	[138]
*MUCINS*/*mucin* expression pathway	Useful therapeutic target	[139,140]

### Discovering associations from BioMAP

The BioMAP tool identifies gene pair associations from a collection of PubMed abstracts using the Vector-Space tf*idf method and a thesaurus consisting of gene terms [41]. Each document, *d*_*i*_, was converted to an M dimensional vector *W*_*i*_, where *W*_*i*_*k* denotes the weight of the *k*^*th*^ gene term in the document and M indicates the number of terms in the thesaurus. *W*_*i*_ was computed using the following equation:

(1) Wik=Tik*logNnk

where *Ti* is the frequency of the *k*^*th*^ gene term in document *d*_*i*_, *N* is the total number of documents in the collection, and *nk* is the number of documents out of *N* that contain the *k*^*th*^ gene term. Once the vector representations of all documents were computed, the association between two genes, *k* and *l*, was computed as follows:

(2) $$association\left[k\right]\left[l\right]=\sum_{i=1}^{N}W_{i}\left[k\right]*W_{i}\left[l\right]$$

where k=1…m and *l* = 1.*m.* This computed association value was then used as a measure of degree of the relationship between the *k*^*th*^ and *l*^*th*^ gene terms. A decision could then be made about the existence of a strong relationship between genes using a user-defined threshold for the elements of the association matrix. Once a relationship was found between genes, the next step was to elucidate the nature of the relationship utilizing an additional thesaurus containing terms relating to possible relationships between genes [41]. This thesaurus was applied to sentences containing co-occurring gene names. If a word in the sentence containing co-occurrences of genes matched a relationship in the thesaurus, it was counted as a score of one. The highest score over all sentences for a given relationship was then taken to be the relationship between the two genes or proteins and was given as:

(3) $$score\left[k\right]\left[l\right]\left[m\right]=\sum_{i=1}^{N}p_{i};\left(p_{i}=1;Gene_{k},Gene_{l},Relation_{m}all\;occur\;in\;sentence_{i}\right)$$

where *N* is the number of sentences in the retrieved document collection, *p*_*i*_ is a score equal to 1 or 0 depending on whether or not all terms are present, *Gene*_*k*_ refers to the gene in the gene thesaurus with index *k*, and *Relation*_*m*_ refers to the term in the relationship thesaurus with index *m*. The functional nature of the relationship was chosen using *arg*_*m*_*scoreklm.* A higher score would indicate that the relationship is present in multiple abstracts.

### Validating associations of BioMAP using Gene Ontology Distance and Protein-Protein Interactions

The TFs obtained from the literature mined data were further annotated using the Gene Ontology for the following six functionalities: TF, TF activator, TF co-activator, TF repressor, TF co-repressor activity, and DNA-binding transcription activity. For all proteins (including TF, kinase, proteins, ligands, receptors, etc.) obtained from the literature-mined data set, we computed its *Gene Ontology Annotation Similarity* (Gene Ontology Distance) with respect to all other proteins in the data.

#### Gene Ontology Annotations Similarity

Each protein pair was evaluated by computing the *Gene Ontology Annotation Similarity*, which was calculated using the Czekanowski-Dice [42] similarity method as follows:

(4) $$d\left(P_{i},P_{j}\right)=\frac{\left[GO\left(P_{i}\right)\Delta GO\left(P_{j}\right)\right]}{\left[GO\left(P_{i}\right)\cup GO\left(P_{j}\right)\right]+\left[GO\left(P_{i}\right)\cap GO\left(P_{j}\right)\right]}$$

where Δ is the symmetric set difference, # is the number of elements in a set, and *GO*(*P*_*i*_) is the set of GO annotations for *P*_*i*_. Similarly, we computed *GO*(*P*_*j*_) for *Pj*. If the *Gene Ontology Annotation Similarity d*(*P*_*i*_*,P*_*j*_) between two proteins was less than 1.0, they were considered to be interacting, thus forming an interaction network. The GO annotations were identified for each protein from UniProt http://www.uniprot.org. We then further scored the interactions in this network using the *protein-protein interaction algorithm* described below.

#### Protein-Protein Interaction Algorithm

Since the available knowledge about protein-protein interactions is incomplete and contains many false positives, a major limitation common to all interaction networks is the quality of the interacting data used. To remove error with respect to false-positives, we developed a *protein-protein interaction algorithm*, which outputs the interaction scores that are annotated on the network as the interaction strength [41,43]. This algorithm consists of six basic steps: (i) identify the protein pair *P*(*i,j*) and its associated structures given in the protein data bank (PDB); (ii) predict the probable interacting residues of each PDB structure in the given pair using the physico-chemical properties of its residues, including hydrophobicity, accessibility, and residue propensity; (iii) compute the distance between the C-alpha coordinates of the probable interacting residues of the given pair; (iv) evaluate the ratio of the number of residues actually interacting with the probable interacting residues based on the distance threshold of C-alpha coordinates; (v) identify the protein pair as interacting or non-interacting based on the given distance threshold; and, (vi) evaluate the interaction of the gene pair - if 30% of the total number of PDB structures for the given protein pair (*i,j*) satisfies the distance threshold, then the pair is considered interacting.

(5) $$ProteinInteractionScore_{i,j}=\frac{\text{\#}ofInteractingResidues}{ProbableNumberOfInteractingResidues}$$

(6) $$InteractionBetween\mathrm{Pr}oteinsScore_{i,j}=\frac{\#ofInteractingPDBstructures}{TotalNumberOfPDBstructures}$$

### Construction of TF interaction network of CRC

The associations satisfying the above Gene Ontology distance and protein-protein interactions criteria were used to construct the TF interaction network of CRC.

#### Determination of network topology

Network topology is an important parameter that defines the biological function and performance of the network [44]. Network properties such as degree, centrality, and clustering coefficients, play an important role in determining the network’s underlying biological significance [45,46]. For the topological analysis, we considered *degree*, *clustering coefficient*, and *betweenness* (centrality). *Degree* is the number of edges connected to node *i*. The *clustering coefficient* of node *i* is defined as $C_{i}=\frac{2n}{k_{i}\left(k_{i}-1\right)}$, where *n* is the number of connected pairs between all the neighbors of node *i*, and *k*_*i*_ is the number of neighbors of *n*. *Betweenness* for node *i* is the number of times the node is a member of the set of shortest paths that connects all pairs of nodes in the network, and it is given as $C_{B}\left(n_{i}\right)=\sum_{j<k}g_{\mathrm{jk}}\left(n_{i}\right)/g_{\mathrm{jk}}$, where *g*_*jk*_ is the number of links connecting nodes *j* and *k*, and *g*_*jk*_(*n*_*i*_) is number of links passing through *i.* These network properties were computed using the igraph package of statistical tool R ( http://www.r-project.org).

### Ranking of TFs using multi-level, multi-parametric features

The TFs were ranked using multi-level, multi-parametric features to better understand their significance in the TF interaction network of CRC. Multi-level refers to the various computational analysis stages that are involved in the detection of the important TFs, as indicated in Figure 1. Multi-parameter features refer to topological and biological parameters and their associated features. Topological parameters can identify relevant nodes in the network; however, annotating the edges with biological parameters (edge strength) will help reveal biologically important nodes in the network.

The edges are annotated using the *Gene Ontology Annotation Similarity Score* and the *Protein Interaction Propensity Score*. As individual edge weights alone cannot capture the complexity of the network [47,48], we also computed the *Gene Ontology Annotation Similarity Score* by considering the average edge weight of each protein and its interacting neighbors [47,48]:

(7) $$GeneOntologyAnnotationSimilarityScore_{i}=\frac{\sum_{i=1}^{N}\sum_{j=1}^{K}{\left(GO\right)}_{i,j}}{K}$$

where *N* is the total number of nodes in the network, *i* is the node in consideration, *K* is the number of immediate neighbors of node *i*, and *j* is the interacting neighbors. The calculation of the *Gene Ontology Annotation Similarity Score* is illustrated in Additional file 1. The *Protein Interaction Propensity Score* for a given node was computed based on the assumption that proteins mostly interact among the domains of their own family [49] and was thus computed as

(8) ProteinInteractionPropensityScorei=∑i=1N∑j=1KProteinInteractionScoreijK∑i=1N∑j=1NProteinInteractionScoreijN

where *N* is the total number of nodes in the network, *i* is the node in consideration, and *K* is the number of immediate neighbors of node *i*. An illustration of the propensity score calculation is shown in Additional file 1.

These methods yielded CRC-relevant nodes in our TF interaction network. We then used node prioritization algorithms to rank the nodes in the network using the following steps:

(a) *Un-weighted and weighted node prioritization*

(i) *Node prioritization based on un-weighted topological and biological features*: In this method, the node prioritization used all four features that were described and computed in the previous steps and was calculated as,

(9) $${\text{NodeStrength}}_{\text{i}}=\frac{\sum_{\text{i=}1}^{\text{N}}{\left(\text{Clust. Coeff.+Betweeness+Gene Ontology Annotation Similarity score+Protein Interaction Propensity score}\right)}_{i}}{4}$$

(ii) *Node prioritization based on weighted topological and biological features*

(10) $$NodeStrength_{i}={\sum_{i=1}^{N}\left[0.4\left(\text{Protein Interaction Propensity Score+}\right)+0.2\left(\text{Clust. Coeff.+Betweeness+Gene Ontology Annotation Similarity score+Protein Interaction Propensity score}\right)\right]}_{i}$$

The actual weights, 0.4 and 0.2, were determined empirically, and the higher weight was associated with the feature *Protein Interaction Propensity Score* since it is a structure-based feature.

#### Validation of proteins and its interaction

Prior to computing the hypergeometric analysis and modules, we validated the proteins and their interactions using KEGG ( http://www.genome.ad.jp/kegg), HPRD [50], and Random Forest classifier of WEKA [51].

(b) * Node-node association prioritization based on hypergeometric distribution*

The basic assumption of hypergeometric distribution is that it clusters the proteins with respect to their functions. That is, if two proteins have a significant number of common interacting partners in the network, then they have functional similarities and therefore also contribute to each other’s expressions [52]. The topological parameter, *betweenness*, finds the centrality of a node in the network. Hypergeometrically-linked associations between two nodes essentially link two nodes that may individually have very high betweenness scores but have low edge weight scores. Additional file 2 describes the advantages of using the hypergeometric distribution metric. This parameter is also essential to identifying those nodes that cannot be identified using standard features.

The nodes with very high *p-values* have higher statistical significance, suggesting that their functional properties play a major role in the network. The *p-value* for each association between two proteins, *P*_*i*_ and *P*_*j*_, was computed as follows:

(11) $$P\left(N,n_{1},n_{2},m\right)=\frac{\left(N-n_{1}\right)!\left(N-n_{2}\right)!n_{1}!n_{2}!}{N!m!\left(n_{1}-m\right)!\left(n_{2}-m\right)!\left(N-n_{1}-n_{2}+m\right)!}$$

where *n*_*1*_ and *n*_*2*_ is the number of interacting proteins of *P*_*i*_ and *P*_*j*_, m is the number of common proteins of *P*_*i*_ and *P*_*j*_, *n*_*1*_ is the total number of proteins interacting with *P*_*i*_, *n*_*2*_ is the total number of proteins interacting with *P*_*j*_, *n*_1_-*m* is the number of proteins that interact only with *P*_*i*_, *n*_2_-*m* is the number of proteins that interact only with *P*_*j*_, and *N* is the total number of proteins in the dataset.

(c) *Construction of functional module*

We defined a module as the sub-graph of a network if it was associated with at least one TF. It is assumed that proteins in a particular module perform similar functions and could be together considered a module for that specific function [53]. For module construction, the nodes with high prioritization scores obtained through the un-weighted and weighted topological and biological features associations and the hypergeometric associations were considered. All direct interactions of the prioritized TFs were used to extract modules.

(d) *TF module ranking*

For the module rankings, each node within the module was annotated with the *Node Strength* obtained using equations (9) and (10). The module score for each of the modules was then computed as

(12) $$AverageModuleScore_{i}=\sum_{j=1}^{C}\frac{NodeStrength_{j}}{C}$$

where, *i* is the *i*^*th*^ module and C=3⋯M*,* where *C* denotes the number of nodes in the module and *M* is the largest module identified in the TF interaction network. The *p-values* were then computed for each TF in the modules as follows [54]:

(13) $$p-value=1-\sum_{i=0}^{k-1}\frac{\left(\begin{array}{c} S\\ I \end{array}\right)\left(\begin{array}{cc} N & S\\ C & I \end{array}\right)}{\begin{array}{c} N\\ C \end{array}}$$

where *S* is the total number of modules present in the TF interaction network of CRC excluding the TF under consideration; *C* is the module size; *N* is the total number of nodes in the whole network; *I* is the number of modules with the specific TF under consideration; and *k* is the module. A module that had TFs with p <0.05 were considered for further analyses.

### Validation by pathway analysis

The functional analysis of the highly ranked TFs and their corresponding modules was calculated using pathways identified by MetaCore^TM^. The *p-values* for these pathways were based on their hypergeometric distributions, which was dependent on the intersection between the user’s data (i.e., associations identified from BioMAP and validated by Gene Ontology distance and Protein Interaction Propensity Score) and the set of proteins obtained from the MetaCore^TM^ database in the pathway, and were computed as:

(14) $$\text{p}-\text{value}\left(\text{r, n, R, N}\right)=\sum_{\text{i}=\max\left(\text{r,R+n-N}\right)}^{\min\left(\text{n,R}\right)}\text{P}\left(\text{i, n, R, N}\right)=\frac{\text{R}!\text{n}!\left(\text{N}-\text{R}\right)!\left(\text{N}-\text{n}\right)!}{\text{N}}\sum_{\text{i}=\max\left(\text{r,R+n}-\text{N}\right)}^{\min\left(\text{n,R}\right)}\frac{1}{\text{i}!\left(\text{R}-\text{i}\right)!\left(\text{n}-\text{i}\right)!\left(\text{N}-\text{R}-\text{n}+\text{i}\right)!}$$

where *N* is the global size of MetaCore^TM^ database interactions, *R* is the user list (identified from BioMAP), *n* is the nodes of *R* identified in the pathway of consideration, and *r* is the nodes in *n* marked by association. The pathways with *p-value* < 0.05 were further analyzed for their functional relevance. This analysis identified the pathways associated with TFs, which could then be experimentally analyzed by biologists in order to validate their associations and importance in CRC.

## Results

### Data collection and pre-processing

We used PubMed abstracts to obtain a global perspective of TFs in the TF interaction network of CRC. For the key list given in Table 1, BioMAP extracted 133,923 articles from PubMed. From these PubMed abstracts, BioMAP identified 2,634 unique molecular entities that were mapped to Swiss-Prot gene names.

### Construction of TF interaction network of CRC

For the 2,634 molecular entities, using the *Gene Ontology Annotation Similarity Score*, we identified 700 gene interactions that involved at least one TF (the network consisted of 117 TFs and 277 non-TFs, for a total of 394 network proteins). Though the bait list had only one TF, the output dataset contained a large number of TFs, indicating the importance of TFs and their roles in CRC. This also demonstrated that bait lists that are highly relevant to the disease of interest can extract a large amount of knowledge from regardless of the vastness of the literature. In addition to the TF interactions, we identified 900 interactions found solely among non-TF entities. Also among the initial 700 interactions 553 interactions were identified in HPRD database.

Among the 394 proteins, only 215 had known protein data bank (PDB) IDs, which produced a total of 3,741 PDB structures (X-ray). Of the initial 700 interactions, 377 interactions were associated with these 3,741 PDB structures. These interactions were evaluated using the previously-described in-house protein-protein interaction algorithm [41,43]. A 6 Å C-alpha distance threshold and 10% threshold for minimum number of interacting residues were initially used to identify interactions between PDB structures; if 30% of structures satisfied these conditions, the protein pair was established to be probably interacting [55,56]. From the 377 interactions, 264 interactions satisfying the 6 Å distance/structure criteria were identified. In these 377 interactions, 278 interactions were validated using HPRD database. These interactions had more than 50% of the interacting residues while the remaining 99 interactions had fewer than 50% of the interacting residues.

**Figure 2:**
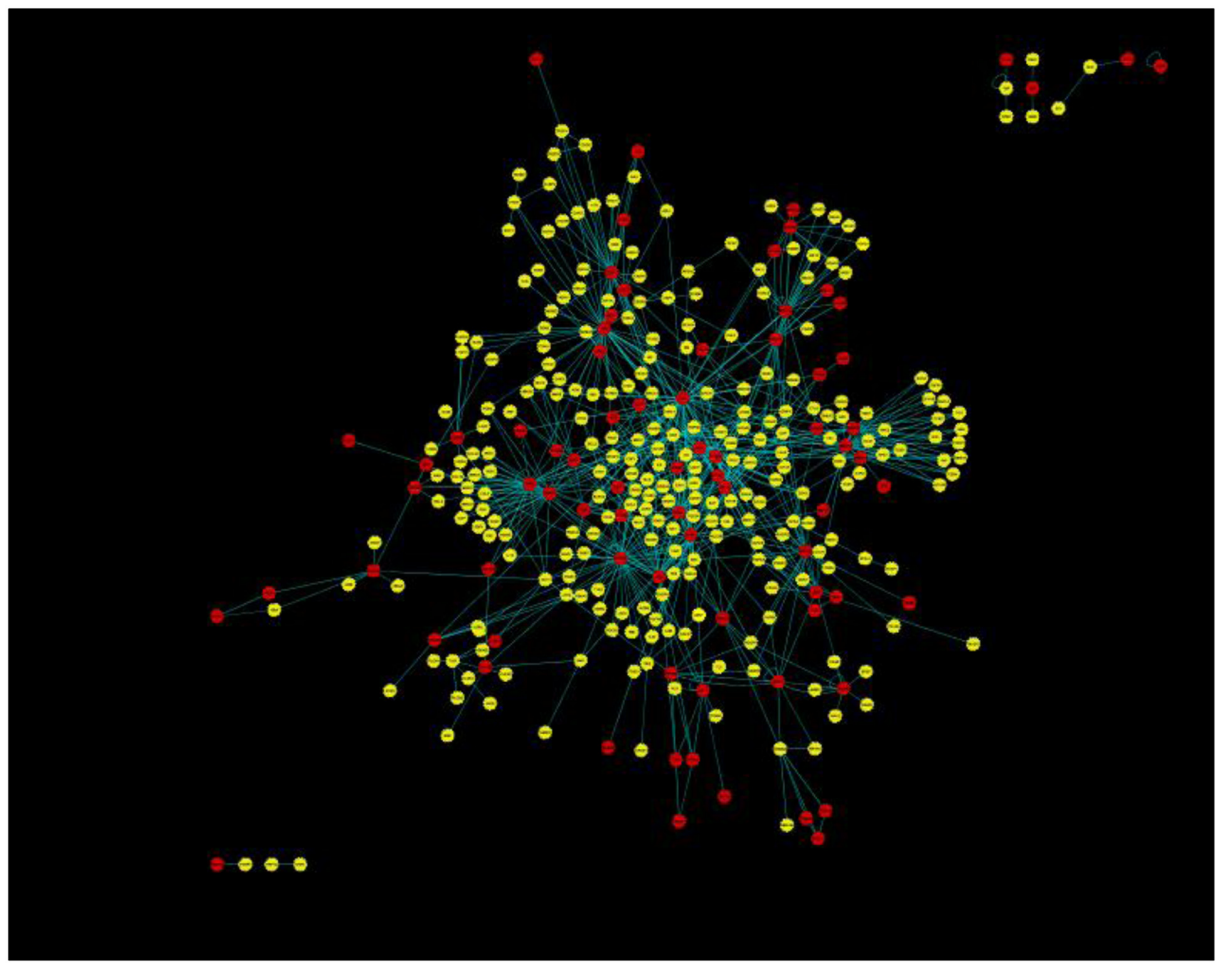
**Transcription Factor Interaction network.** The red nodes indicate transcription factors while yellow represents the remaining proteins.

In the constructed TF interaction network for CRC, shown in Figure 2, the edges were annotated with the *Gene Ontology Annotation Similarity Scores* and *Protein Interaction Propensity Scores* (computations are depicted Additional file 1)*.*

### Topological analysis of the TF interaction network of CRC

In the TF interaction network shown in Figure 2, the node degree ranged from 0 to 48, with an average degree of 4.29. A total of 133 nodes were identified with *betweenness* measures (i.e., these nodes passed through the paths of other nodes), and 149 nodes were identified with *clustering coefficient* measures. Table 2 lists the top 19 nodes identified using *degree*, *clustering coefficient*, and *betweenness*. In addition to identification of the TFs with the highest topological feature scores, other proteins with similar topological rankings were also identified. All the nodes in the network were annotated with these topological parameters.

**Table 2:** Top ranked nodes identified for each of the topological parameters

**Metric**	**Top 20 ranked proteins**
*Degree*	*p53* (48), *c-Jun* (48), *STAT3* (41), *NF-kB-P65* (36), *ESR1* (35), *NF-kB/TNFRSF11A* (33), *SMAD3* (33), *SP1*(32), *STAT1* (32), *DAND5* (31), *c-Myc* (30), *E2F1* (28), *SMAD2* (26), *MEF2A* (26), *RARA* (24), *GCR* (23), *SMAD4* (20), *HIF1A* (18), *MEF2C* (18)
*Clust. Coeff*.	*p53*, *Akt1*, *STAT3*, *RARA*, *E2F1*, *STAT1*, *c-Jun*, *NF-kB-P65*, *CREM*, *Elk-1*, *c-Myc*, *SMAD3*, *Lef1*, *HIF1A*, NF-*k*B/*TNFRSF11A*, *ESR1*, *GCR*, *PPARA*, *MEF2A*
*Betweenness*	*p53*,*c- Jun*, *STAT3*, *c-Myc*, *STAT1*, *RARA*, *ESR1*, *NF-kB-P65*, *SMAD3*, *E2F1*, *Akt1*, *MEF2A*, *NF-kB/TNFRSF11A*, *MK14*, *SP1*, *DAND5*, *EP300*, *GCR*, *JAK2*

### Ranking of TFs using multi-level, multi-parametric features

#### Node prioritization un-weighted/weighted schema (using topological and biological features)

The topological and biological features – *betweenness*, *clustering coefficient*, *Gene Ontology Distance Score*, and *Protein Interaction Propensity Score* – were computed for the 394 nodes in the interaction network (Figure 2). Nodes were ranked using the node strength, which computed using both weighted and un-weighted scoring schemes (discussed in the methods section); Table 3 shows the top 10 TFs for each scoring schema.

**Table 3:** Ten top-ranked nodes identified by each weighting scheme

**Schema**	**Top 10 nodes**
**Un-weighted**	p*53*, *c-Jun*, STAT3*, ABL1, c-Myc, GLI1, CDC6, RARA, STAT1, ESR1*
**Weighted**	*p53*, *ABL1*, *c-Jun*, *GLI1*, *STAT3*, *NF-κB*, *PIAS1*, *c-MYC*, *ESR2*, *MK11*

#### Validation of proteins and their interactions

Proteins and their interactions were validated using KEGG, HPRD, and Random Forest. The proteins in each interaction were validated using KEGG pathways and the HPRD cancer signalling pathways. If a protein was present in the KEGG colon cancer pathways, it was annotated as HIGH. If a protein was in KEGG cancer pathways or HPRD cancer signalling pathways, it was annotated as MEDIUM. If a protein was not present in any of the above pathways but in other pathways of KEGG, it was annotated as LOW. In the initial 700 interactions, there were 20 proteins associated with CRC, 183 proteins associated with KEGG cancer pathways/HPRD cancer signalling pathways, and 128 associated with other KEGG pathways. Interactions were annotated as HIGH if both proteins were annotated HIGH or a combination of HIGH-MEDIUM or HIGH-LOW; MEDIUM if both proteins were annotated MEDIUM or MEDIUM-LOW; and LOW if both proteins were annotated LOW.

#### Node prioritization using hypergeometric distribution

Table 4 shows the top 10 TF associations with the *p-value* < 0.05.

**Table 4:** Ten top-ranked TF associations with significant *p-values* (< 0.5)

**TFs association**	***p-value***
*ESR1*: *CCND1*	1.5E-63
*NF-kB*: *NF-kb-p65*	6.13E-42
*SMAD2*: *CBP*	9.25E-23
*MEF2A*: *MEF2D*	1.145E-21
*SMAD3*: *SMAD2*	1.94E-16
*SMAD2*: *SMAD4*	2.92E-13
*c-Jun* : *GCR*	9.72E-8
*RXRA*: *NCOR1*	1.04E-6
c-JUN: *ESR1*	2.23E-6
*ESR1*: *SP1*	1.56E-5

#### Modules analysis

For each of the TFs in the TF interaction network (Figure 2), functional modules of size greater than or equal to three nodes were identified. This process yielded 70 modules with 3 nodes, 35 modules with 4 nodes, 18 modules with 5 nodes, 12 modules with 6 nodes, and 56 modules with 7 or more nodes. Each module was then analyzed using the average module score (equation (12)), and the significance of the TFs in each of these modules was assessed at *p* < 0.05 (equation (13)). Tables 5 and 6 show the TFs identified in top-scored modules and bottom-scored modules for the two scoring schemas, respectively.

**Table 5:** TFs identified in top 10 modules

**Schema**	**Nodes**	**TFs identified**
Un-weighted	3	*p53*, *E2F1*, *STAT3*, *STAT1*, *MEF2A*
	4	*p73*, *c-Jun, NF-kB-P65, p53, STAT3, NF-kB/TNFRSF11A, ETS1, ETS2, E2F1, c-Myc, SMAD3*
	5	*ESR1, c-Jun, SP1, DAND5, MEF2C, GCR, GRIP1*, *RARA*
	6	*STAT3, c-Myc, p53, SMAD3, STAT1, NF-KB/TNFRSF11A, ESR1, NF-kB-P65, SP3, IRF1*
Weighted	3	*E2F1, p53*
	4	*p73, c-Jun, NF-kB/TNFRSF11A, NF-kB-P65, STAT3, ETS1, ETS2, c-Myc*
	5	*DAND5, ESR1, c-Jun, SP1, MEF2C, GCR, RARA, GRIP1, NRSF*
	6	*STAT3, c-Myc, p53, SMAD3, STAT1, NF-kB/TNFRSF11A, ESR1, NF-kB-P65, SP3, ATF2, Elk-1*

**Table 6:** TFs associated with bottom 3 modules

**Schema**	**Nodes**	**TFs identified**
Un-weighted	3	*REST, ITF2, TF7L2, Elk-1, GATA-1, SRF*
	4	*FOXA1, FOXA2, FOXA3, GLI1, GLI2*
	5	*ESR2, ITF2, TF7L2, Lef1, REST, c-Myc, PPARD, SLUG*
	6	*CREB1, c-Jun, DAND5, SP1, SP3, TNF11, HAND1, VDR, STAT1, STAT3*
Weighted	3	*GATA-1, ITF2, REST, TF7L2, SRF, Elk-1*
	4	*GLI1, GLI2, FOXA1, FOXA2, FOXA3*
	5	*ESR2, ITF2, Lef1, c-Myc, PPARD, REST, TF7L2*
	6	*CREB1, c-Jun, DAND5, SP1, SP3, NF-kB/TNFRSF11A, NF-kB-P65, HAND1, STAT3, STAT1, VDR, KPCA*

### Validation using pathway analysis

For the bait list given in Table 1, literature mining identified an additional 2,634 entities which were then analyzed for their relevance in CRC pathways. The significance of the literature-mined molecules with respect to TFs, ranked TFs, functional modules, and their associated functional pathways was determined using MetaCore^TM^ from GeneGO. The MetaCore^TM^ tool identified 39 significant pathways for the bait list data with *p-values* ranging from 3.591E-10 to 7.705E-3. However, when augmented with literature-mined molecules, MetaCore^TM^ identified 286 significant pathways with *p-values* ranging from 1.253E-17 to 2.397E-2. These 286 pathways were analysed for their functional groups and were classified as major if associated with more than 3 pathways, or minor, if associated with 3 or fewer pathways. The 286 pathways identified were classified in 13 major functional groups and 6 minor groups.

## Discussion

### Global analysis of TF interaction network of CRC

In the TF interaction network (Figure 2), all 700 interactions were identified using the *Gene Ontology Annotation Similarity Score*. However, only 264 interactions out of 700 interactions could be further scored by the *Protein-Protein Interaction* method. Protein-protein interaction criteria is significant as it has a greater probability of revealing an *in-vivo* interaction of functional importance [43,44,55,56]; the protein-protein interaction algorithm is built on structure data, and structure provides the basis of protein functionality.

We observed that a multi-parametric approach using both *Gene Ontology Annotation SimilarityScore* and *Protein Interaction Propensity Score* can help identify CRC-relevant interactions that may not have been identified if only one of the methods was used for construction of the TF interaction network. For example, when only the *Gene Ontology Annotation Similarity Score* was used, interactions between ATF2_HUMAN and MK01_HUMAN (MAPK1, ERK) or ELK1_HUMAN and MK08_HUMAN (JNK1) were either scored very low or missed all together. The interaction between ATF2-MK01 was identified only in the cellular function (0.6), but not in the molecular function, when the *Gene Ontology Annotation Similarity Score* was calculated. However, using the *Protein Interaction Propensity Score*, this interaction was scored high (0.74) as compared to cellular and molecular function. This interaction would also have been missed if only the molecular function for the *Gene Ontology Annotation Similarity Score* was used.

Similar observations were made for ELK1_HUMAN and MK08_HUMAN (JNK1), which had *Gene Ontology Annotation Similarity Scores* of 0 for cellular function, 0.67 for molecular function, and 0 for biological process, but had a P*rotein Interaction Propensity Score* was 0.25. The *MAPK* pathway, which is known to be important in CRC [57-59], is not well established in literature with respect to *ATF2* and *MK01* interaction. Similarly, *ELK-1* and *JNK* isoforms are known separately as cancer relevant genes regulating important oncogenic pathways, such as cell proliferation, apoptosis, and DNA damage; however, their possible interactions and biological consequences in the context of CRC have not been reported [60]. The identification of this possible interaction then illustrates the benefit of augmenting literature data with both *Gene Ontology Annotation Similarity* and *Protein Interaction Propensity Scores*, which increases the probability of revealing novel interactions, ultimately resulting in a larger network perspective on CRC.

### Topological network analysis

All the nodes in the interaction network shown in Figure 2 were evaluated based on three topological features: *degree*, *betweenness*, and *clustering coefficient* respectively. As shown in Table 2, *p53*, *c-Jun*, *c-Myc*, *STAT3*, *NF-kB-p65*, *NF-kB/TNFRSF11A*, *SMAD3*, *SP1*, *STAT1*, *E2F1*, *MEF2A*, and *GCR* were highly scored with respect to all three features. On the other hand, *SMAD2*, *SMAD4*, *Elk-1*, *Lef1*, *CREM*, *EP300*, *JAK2*, *Akt1*, *PPARA*, and *MK14* were scored by only one of the three topological features. This type of topological stratification can provide a strong triaging basis before further experimental validation.

The top ranking nodes were further analysed for their significance in CRC using literature evidence. For example, *p53*, which had a maximum degree of 48 and also scored highly on the other two parameters, is known to be involved in pathways important in CRC in addition to having \prognostic value [61,62]. In the case of *c-Jun*, its activation by *JNK* is known to be critical for the apoptosis of HCT116 colon cancer cells that have been treated by *curcumin*, an herbal derivative with anti-cancer properties [63,64]. Another important molecule identified was *STAT3*, which is a key signalling molecule responsible for regulation of growth and malignant transformation. *STAT3* activation has been shown to be triggered by *IL-6*, and a dominant negative *STAT3* variant impaired *IL-6*-driven proliferation of CRC cells *in vitro*[65-67]. Other examples of TFs with high node scores within the TF interaction network of CRC are shown in Table 2. Analysis of these results shows that a majority of the TFs identified using literature augmented data and scored using topological methods are known to be highly relevant with respect to CRC.

### Ranking transcription factors using multi-level, multi-parametric features

On comparing the results of un-weighted and weighted feature analysis methods, as shown in Table 3, it can be seen that six of the top ten nodes*, p53*, *c-Jun*, *STAT3*, *ABL1*, *c-Myc*, and *GL11*, were common to both. Comparison of the nodes obtained using only the topological features (Table 2) with those nodes obtained using both topological and biological features (Table 3)revealed that eight nodes were common to both: *p53*, *c-Jun*, *STAT3*, *c-Myc*, *RARA*, *STAT1*, *ESR1*, and *STAT3*. The unique nodes identified based on both features in Table 3 were *ABL1*, *GL11*, *CDC6*, *ESR2*, *MK11*, and *PIAS1*. Recent studies have identified *GLI1* as highly up-regulated and *PIAS1*as down-regulated in CRC [68-71]. There is no report so far on association of *ABL1* with CRC, though *BCR-ABL1* is the well-known, clinically-relevant drug target in chronic myelogenous leukema [72]. These analyses resulted in the identification of additional and important TFs that underscore the importance of using a multi-level, multi-parametric approach for ranking TFs.

#### Validation of proteins and its interaction

More than 60% of the proteins in the interactions were associated with KEGG colon cancer pathways, KEGG cancer pathways, or HPRD cancer signalling pathways. This indicates the relevance of the constructed network with respect to cancer. Additionally, 55% of the interactions were annotated as HIGH, 35% as MEDIUM and 10% annotated as LOW, indicating the relevance of the network with respect to CRC. After annotating with HIGH, MEDIUM, and LOW, a Random Forest classifier was used to elucidate the significance of the networks. The precision/recall for the weighted schema was 0.75 and 0.742 respectively, while for un-weighted, it was 0.63 and 0.57 respectively. The ROC for weighted schema was as follows: HIGH = 0.957, MEDIUM = 0.835 and LOW = 0.82. These ROC scores suggest that the multi-parameter approach that was developed can help to identify relevant TFs in the TF interaction network of CRC.

The second node prioritization method, using hypergeometric distribution, helped identify functional associations of the TF nodes within the TF interaction network of CRC. Using this method, 83 associations with *p-value* < 0.05 that involved 26 unique TFs were identified. Table 4 shows the 10 highly-scored associations along with their *p-values*. When compared with the results from Table 2 and Table 3, the hypergeometric distribution method identified nine additional TFs: *ATF-2*, *ETS1*, *FOS*, *NCOR1*, *PPARD*, *STAT5A*, *RARB*, *RXRA*, and *SP3*.

These TFs were then analyzed using the literature in order to confirm any association with CRC. We found that many of these TFs have not been extensively studied in CRC, if at all. *ATF-2* stimulates the expression of *c-Jun*, *cyclin D*, and *cyclin A*, and it is known to play a major oncogenic role in breast cancer, prostate cancer, and leukemia [73]. However, little is known with respect to the role of *ATF-2* in CRC, except for a recent study that identified *ATF-2* over-expression associated with *ATF-3* promoter activity in CRC [74]. Similarly sporadic evidence supports the notion that *PPARD* and *PPAR-δ* are linked to CRC [75,76]. However, several others in the list have not yet been shown to be important in CRC. For example, *RXRA*/*RARA*, the ligand dependent TFs, have not been directly associated with CRC, but have been found to be associated in the network with *PPAR s*, which in turn has been linked to CRC. The *MEF2* family of TFs, which are important regulators for cellular differentiation, have no known direct association with CRC, but *MEF2* is known to associate with *COX-2*, whose expression plays an important role in CRC. *MEF2* is activated by the *MAPK* signalling pathway, along with activation of *Elk-1*, *c-Fos*, and *c-Jun*. Activation of the latter pathways have been shown to contribute to hormone-dependent colon cancer [77]. It appears that the hypergeometric distribution analysis has identified a new group of TFs of potential importance to CRC by virtue of their interaction with genes that are known to play an important role in CRC, although these TFs themselves are not known to have any direct role in CRC.

#### Module analysis

As stated earlier, proteins that are affiliated within a module are more likely to have similar functional properties [52]. For this analysis, the modules considered were sized in the range of 3 and above. This larger module size identified low connectivity nodes which otherwise would have been missed using only the topological, hypergeometric analysis or smaller modules (i.e., only 2 or 3 nodes).

Table 5 shows the TFs that were associated with the 10 highest-ranked modules, all of which had *p-values* < 0.05 (from equation (13)). Table 6 shows the TFs identified in the bottom ranked 5 modules. Twenty TFs were common among the 10 top ranked modules. The five TFs unique between the two scoring schemas were: *MEF2A*, *SP3*, *IRF1*, *ATF-2*, and *Elk-1*. *IRF1*, *SP3* and *ATF-2* were additionally not identified as high-scoring TFs in Table 2, 3, and 4. *IRF1* was identified among the top scoring modules in association with *PIAS1*, *SP3*, and *HIF1A*. Of these associations, *HIF1A* over-expression along with *PIAS1* has been studied amd identified to be associated with CRC. *HIF1A* has also been associated with poor prognosis, and it is currently under consideration as potential biomarker [78].

This module-level analysis also identified many new TFs associated in the lower-scoring modules. The TFs associated with the lower scoring modules listed in Table 6 include *VDR*, *HAND1*, *GLI1*, *GLI2*, *PPARD*, *Lef1*, *FOXA2*, *GATA-1*, *REST*, *ITF-2*, *TF7L2*, and *SLUG*. Out of this group, *GATA-1* presents an example as a novel TF with a possible link to CRC. The loss of expression of the *GATA* family is associated with several cancers; loss of expression for *GATA-4* and *GATA-5*, in particular, have been reported in CRC [79]. No literature evidence is available for the relationship between *GATA-1* and CRC, but our analysis warrants further study in this direction. Similar analysis and follow-up experimental validation of all the remaining TFs identified in both the high- and low-scoring modules can improve understanding of their relevance with respect to CRC.

**Figure 3:**
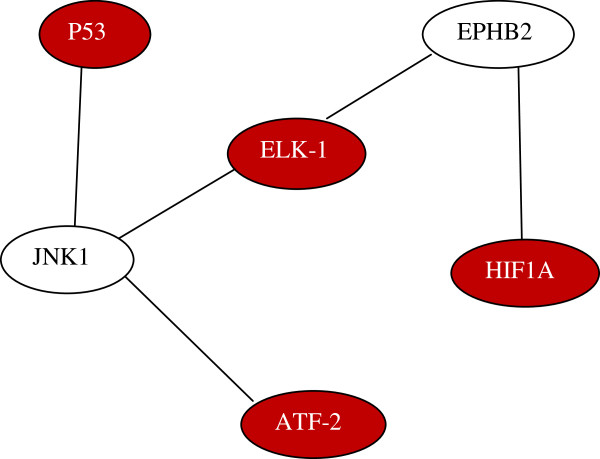
The novel, highly-scored functional module identified shows the association of ELK-1:JNK1 and EPHB2:HIF1A.

Further analysis of high-scoring modules showed that the 3-node modules were mainly associated with p53, particularly via *E2F1*. The 4-node modules were ranked highly when the TFs *c-Jun*, *p53*, and *NF-kB-p65*, all of which are known to be highly relevant to CRC, were present. One of the highly scored 6-node modules was associated with *ATF-2*:*p53*:*JNK1*:*Elk-1*:*EPHB2*:*HIF1A* (Figure 3). *EPHB2* has been associated with the Ras pathway, which in turn is a prominent oncogenic driver in CRC [80], while *Eph* receptors have been identified to be important in CRC [81], though more studies are necessary for better understanding their specific role in CRC. HIF1A over-expression is linked to serrated adenocarcinomas, a molecularly distinct subtype of CRC [82].

Also noteworthy among the 6-node modules is the interaction between *Elk-1* and *JNK* (*Jun N terminal kinase*) isoforms (*MK09* and *MK10* are *JNK2* and *JNK3*, respectively), as there are many promising potential links between *JNK* isoforms and CRCs. These potential links include the established roles of *JNKs* in the development of insulin resistance, obesity, and Crohn’s disease [83], all of which are well-known pre-disposing factors for CRC [84]. The *JNK1* isoform promotes cancers of the liver, stomach, skin, and ovary [85,86], so it is plausible that other isoforms may also be involved in cancer. One of these isoforms, *JNK2*, is known to regulate breast cancer cell migration [87] and has been reported to play a dual role (both tumor promotion and suppression) in liver cancer [88].

The *JNK* interacting partner, *Elk-1*, is one of the critical downstream components of the *Ras-MAPK* pathway, but efforts to target this pathway using *Ras* or *MEK* inhibitors have failed to produce clinical benefits in CRCs and many other types of cancers [89]. One logical explanation for this lack of clinical efficacy is the existence of one or more compensatory mechanisms to ensure the activation of same downstream component, in this case *Elk-1*, and related TFs. *JNK* is known to phosphorylate *Elk-1* on the same site as *ERK1/2* and *Ser-383*, allowing for regulation of its transcriptional activation function [90]. The consequence of *JNK-*induced *Elk-1* activation is not completely clear, but it is known to play a role in cell proliferation and differentiation [91,92]. *Elk-1* and *JNK* isoforms are known cancer-relevant genes that separately regulate important oncogenic pathways, including cell proliferation, apoptosis, and DNA damage pathways [83,93]. Both *Elk-1* and *JNK* have been established as important drug targets in cancer, though not in CRC, and have multiple drugs/inhibitors that are in various phases of clinical trials [85,89]. Therefore, it is plausible that an active *JNK-Elk-1* pathway in CRC could potentially confer resistance to *Ras* or *MEK* inhibitors, presenting a new drug targeting strategy.

A third example of CRC-relevant TFs identified via the methodology used in this paper is *GATA-1*, which was identified in the 5-node module along with *RUNX1SP1*. Recent studies have shown the association of *RUNX1* and *RUNX2* with *TGF*-beta signalling pathways in colorectal cancer [94], suggesting a potential association of *GATA-1* with CRC through *RUNX1SP1*. Our module analysis also revealed several less-studied TFs and their associations in CRC that may be of interest for future studies. These include *IRF1* and *STAT3* in the 5-node module, as well as *Bcl-2*’s associations with 5 different TFs (*STAT3*, *NF-kB*, *ESR1, p53*, *NF-kB-p65*) in the 6-node module.

These analyses show the advantages of using a multi-level, multi-parametric feature for analysing TFs of importance both in CRC and in other diseases. As each of the analysis processes employs different criteria for ranking, biologists will have greater, knowledge-driven power to identify and select targets for further validation.

### Validation using pathway analysis

**Figure 4:**
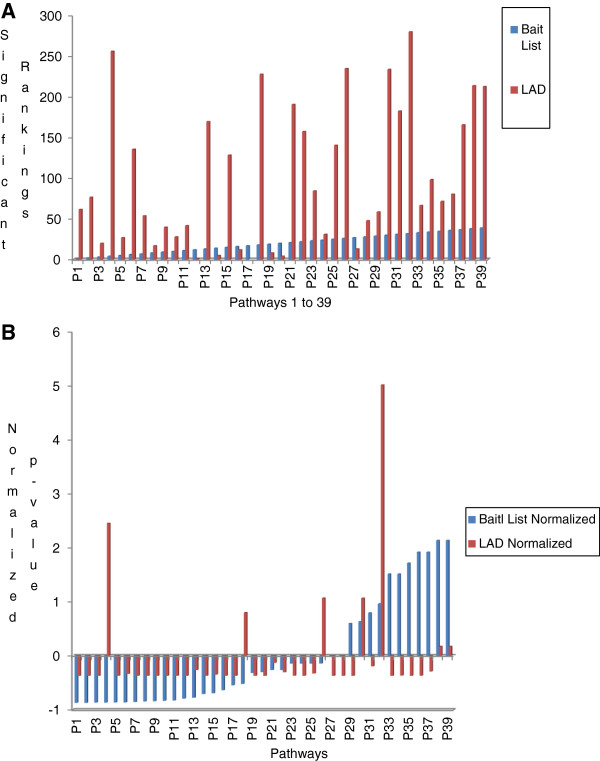
**A Ranking comparison between the Bait list pathways and Literature Augmented Data pathways. B**: *p-value* comparison between the Bait List pathway and Literature Augmented Data pathways.

To better understand the significance of the highly-ranked TFs, modules, and the overall TF interaction network, all 2,634 proteins (output from BIOMAP) were analysed using MetaCore^TM^ for their significance in various pathways from the original bait list (39 pathways) and the literature augmented data-generated list (286 pathways). Figures 4A and B show the comparisons between the rankings and *pvalues* of the bait list and the literature augmented pathways. For analytic purposes, the 286 pathways were further classified according to their functional groups as given by MetaCore^TM^. Table 7 shows the frequency distribution of these pathways with respect to their functional groups. From Table 7 it can be observed that the top three functional groups were Development, Immune Response, and Apoptosis and Survival, which are well-known in CRC. Chemotaxis, which is also listed in Table 7 as associated with four pathways, is the unidirectional movement of a cell in response to any given chemical gradient, which plays an important role in innate and acquired responses. The four chemotaxis-associated pathways were the CXR4 signalling pathway, inhibitory action of IL-8 and leukotriene B4-induced neutrophil-migration, and leukocyte and chemotaxis, all of which have been associated with CRC in literature [95,96], as well as Lipoxin inhibitory action of fMLP-induced neutrophil chemotaxis pathway. This last pathway has not been well-studied in CRC, though lipoxins are known to be associated with anti-inflammatory and proresolving mediators in CRC [97]. The analysis of the chemotaxis functional group demonstrates that while using a small bait list or list of experimental proteins may not fully depict the global profile of a disease, using literature augmented data can help to expand this profile and further help to understand new pathways with respect to disease.

**Table 7:** Relationship between functional groups and number of pathways (13 major functional groups with >3 pathways and 6 minor functional groups with ≤3 pathways) Total Number of Pathways = 286

**Functional groups**	**Number of pathways**
Development	75
Immune response	59
Apoptosis and survival	23
G-protein signaling	18
Transcription	16
Cell cycle	14
Cell adhesion	11
Cytoskeleton remodeling	11
DNA damage	8
Signal transduction	6
Translation	6
Muscle contraction	5
Chemotaxis	4
Other small functional groups	14

It is possible that functional grouping shows a greater preponderance of pathways in areas where TFs appears to be the major mode of regulation (e.g., development, immune response, and survival) and lower prevalence of pathways in areas where post-transcriptional mechanisms play major regulatory role (e.g., signal transduction, DNA damage, and cytoskeleton regulation) due to the text mining process’s focus on ‘transcription factors’. Nonetheless, the top three functional groups are all primarily responsible for general cell fate determination, and deregulation of all these pathways is known to be the underlying basis of oncogenesis.

### Global analysis of TFs in CRC pathways

**Figure 5:**
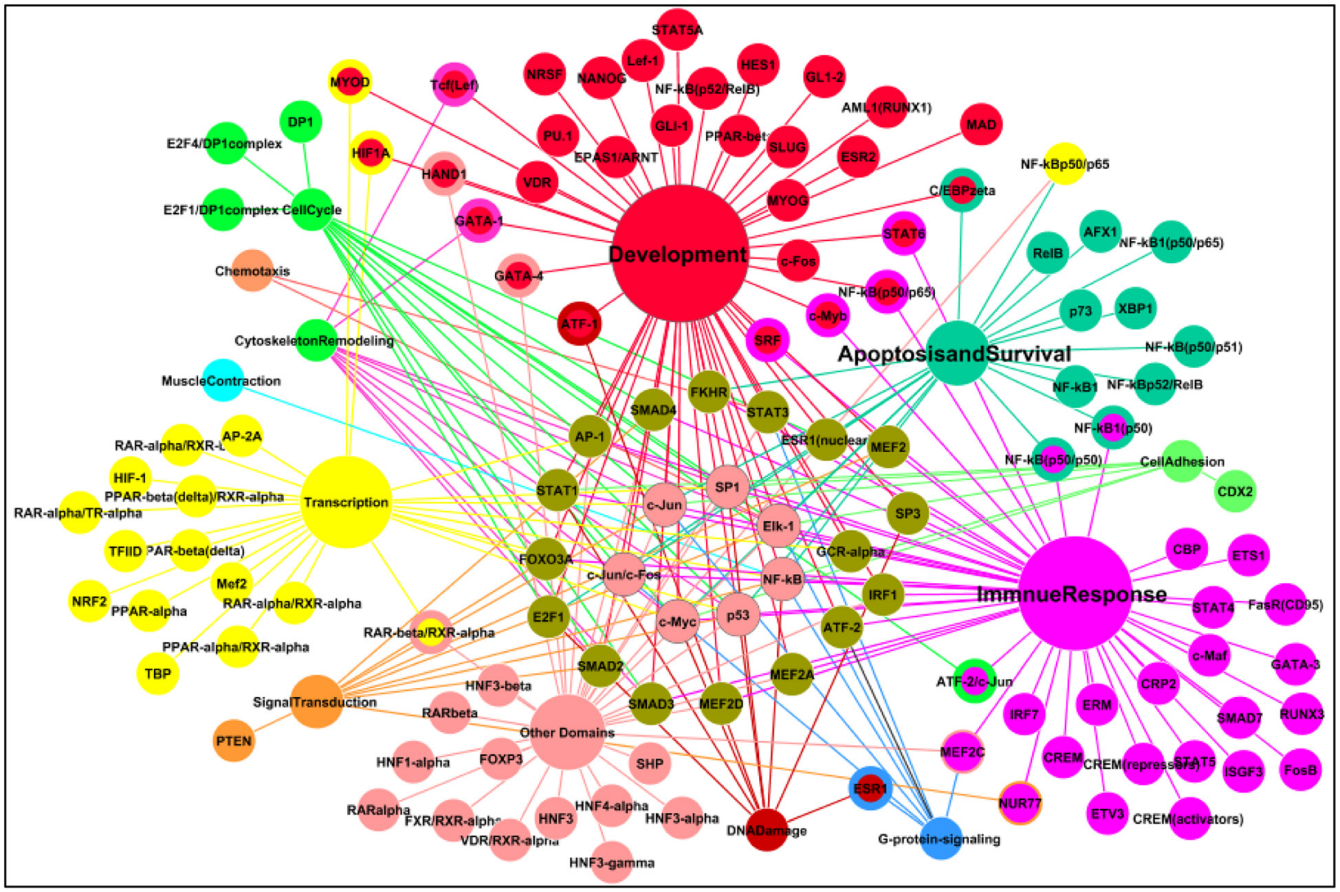
**Functional groups and associated transcription factors. The centermost transcription factors are associated with multiple functional groups.** The size of the functional group represents the relative number of pathways and transcription factors associated with it.

Figure 5 shows the TF distribution profile in each functional group for which the connectivity profile was analyzed. The Development, Immune Response, Transcription, and Apoptosis and Survival functional groups were associated with the highest number of TFs (54, 48, 24, and 20, respectively), whereas the Chemotaxis and Muscle Contraction functional groups were associated with 2 and 1 TFs, respectively. The most highly-ranked TFs identified through the analysis, *p53*, *c-Jun*, and *c-Myc*, were identified in multiple functional groups. TFs such as *RARA*/*RXRA*, *VDR*, and *GATA*, which are specific to certain functional groups, were identified in our ranking analysis as well.

The global analysis that was carried out in this work provides a distinct advantage by enabling the visualization of all network TFs at a glance. It can be seen that the highest connectivity TFs varied from one functional group to another - *STAT3* had 39 connections in Development, *p53* had 26 connections in DNA Damage, (iii) *c-Jun* had 12 connections in Apoptosis and Survival, (iv) *GATA-1* had 5 connections in Cytoskeleton Remodeling, and (v) *c-Myc* had 2 connections in Cell Adhesion. Though *c-Myc* was not identified with very high connectivity in any one functional group, it was present in almost every functional group (and also as a prioritized TF). Additional files 3, 4 and 5 provide the Gene Ontology molecular function and hub nodes for all the functional groups and the connectivity profile order of the TFs in each functional group.

Table 8 shows the highly scored modules that were analysed with respect to their associated functional groups, pathways and GO Terms From this table it can be observed that the modules identified belonged mostly to the Apoptosis and Survival, Immune Response, DNA Damage, Development, and Transcription functional groups. Microsatellite instability due to defective DNA repair pathways and impairment of pathways that are developmentally conserved (e.g., Wnt/beta-catenin pathway) are the key molecular drivers of CRC origin, validating the significance of identifying the DNA Damage functional. Moreover, three of the modules were also associated with pathways are specific to inflammation, providing new clues to possible mechanisms for the widely accepted CRC-predisposing effect of inflammation. Thus the approach we developed not only validated some of the well-established paradigms of CRC biology but also provided actionable clues to yet-unstudied potential mechanisms. From this table it can be concluded that our methodology was able to reveal TFs that are already proven to be prognostic, those are under on-going studies for verifying prognostic values, and novel ones that can be further studied. Additional file 6 gives the profile of the prognostic values for more TFs not included in Table 8.

**Table 8:** Analysis of 5 highly-scored modules in each size category, with respect to functional groups and pathways, using MetaCore^TM^ from GeneGO

**Module**	**Functional groups**	**Pathway (*p-value*)**
**Module Size: 3**		
1. *CHK2:p53:E2F1*	Apoptosis and Survival	DNA-damage-induced apoptosis (1.63E-6)
2. *ATR:p53: E2F1*	DNA Damage	*ATM*/*ATR* regulation of G1/S checkpoint (5.7 E-8)
	Apoptosis and Survival	DNA-damage-induced apoptosis (1.63E-6)
3. *APEX1:HIF1A:p53*	Transcription	Role of *AKT* in hypoxia *HIF1* activation (1.63E-9)
4. *IL-22:STAT3:STAT2*	Immune Response	*IL-22* signalling pathway (4.51E-6) *(****Inflammation****)*
5. *IL-9R:STAT1:STAT3*	Immune Response	*IL-9* signalling pathway (1.64E-5)
**Module Size: 4**		
1. *COX-2:NF-kB:p53*: *NF-kB-p65*	Immune Response	*MIF* in innate immunity response (1.48E3) (***Inflammation***)
2. *TNFA: c-Jun*: *NF-kB:NF-kB-p65*	Apoptosis and Survival	*TNFR1* signalling pathway (2.44E-14)
3. *p53:c-ABL:c-Jun*: *p73*	Apoptosis and Survival	*p53* dependent apoptosis (7.67 E-9)
4. *ETS2:ETS1:c-Jun*: *c-Myc*	Immune Response	*ETV3* effect on *CFSI* promoted macrophage differentiation(1.04E-5)
5. *MAPK11:MEF2C*: *MEF2A:c-Jun*	Immune Response	Function of *MEF2* in T lymphocytes (0.0003)
		*TLR*-signalling pathways (8.63E-10) (***Inflammation***)
**Module Size: 5**		
1. *BCLX:DAND5:ESR1*: *c-Jun:SP1*	Development	Prolactin receptor signalling (3.52E-10)
	DNA Damage	Role of *Brca1* and *Brca2* in DNA repair (8E-13)
2. *TCF7L2:Lef1:c-Myc*: *PPARD:NRSF*	Development	*Wnt* signalling pathway (2.45E-11)

## Conclusions

The text mining approach developed in this paper was able to correlate known and novel TFs that play a role in CRC. Starting with just one TF (SMAD3) in the bait list, the literature mining process was able to identify 116 additional TFs associated with CRC. The multi-level, multi-parametric methodology, which combined both topological and biological features, revealed novel TFs that are part of 13 major functional groups that play important roles in CRC. From this, we obtained a novel six-node module, ATF2-P53-JNK1-ELK1-EPHB2-HIF1A, which contained an association between JNK1 and ELK1, a novel association that potentially be a novel marker for CRC.

The approach identified new possibilities, such as *JNK1*, for targeted CRC therapies using inhibitors that are undergoing clinical trials for non-cancer indications. Furthermore, pending further validation, some of the genes identified by our approach with possible new links to CRC may well prove to be new biomarkers for drug response and prognosis in CRC. For further follow-up, we plan to work on multiple bait lists, annotate the text mining data with gene expression, identify the gene signatures for the known and novel pathways, use in-vitro model validation, and, ideally, develop clinical trials.

## Abbreviations

(CRC): Colorectal cancer; (TFs): Transcription factors; (TF): Transcription factor.

## Competing interests

The authors declare that they have no competing interests.

## Authors’ contributions

MPP: conceptualizing and developing methodology, data collection, writing and analysis of all the algorithms, writing manuscript, NKAP: critical analysis of the manuscript, valuable input as cancer specialist, writing of the manuscript, MJP: PI of the project, conceptualizing the objective, writing manuscript, valuable inputs at all the time. All authors read and approved the final manuscript.

## Pre-publication history

The pre-publication history for this paper can be accessed here:

http://www.biomedcentral.com/1471-2407/12/331/prepub

## Supplementary Material

Additional file 1 Gene Ontology Annotation Similarity Score Protein-protein interaction algorithm. Click here for file

Additional file 2 Hypergeometric distribution. Click here for file

Additional file 3 Few transcription factors and their associated Gene Ontology molecular functions. Click here for file

Additional file 4 **Nodes with highest number of connections identified for each functional group (defined by MetaCore**^**TM**^**in GeneGO).** Click here for file

Additional file 5 **Functional group transcription factor distribution.** Transcription factors are arranged in decreasing order with respect to their connectivity in each functional group. Click here for file

Additional file 6 Analysis of transcription factors identified with prognostic value in CRC. Click here for file
