# Supplementary material for: A systems biology approach to the global analysis of transcription factors in colorectal cancer
Source: BMC Cancer. 2012 Aug 1;12:331. doi: 10.1186/1471-2407-12-331 (PMC3539921; doi:10.1186/1471-2407-12-331)
Supplement: Additional file 1 — Gene Ontology Annotation Similarity Score Protein-protein interaction algorithm. [file 1471-2407-12-331-S1.docx]

**Additional File I**

**Gene Ontology Annotation Similarity**

**And**

**Gene Ontology Annotation Similarity Score**

Protein Cellular Region

GLI1 Nucleus, cytoplasm

GLI2 Nucleus

KAPCA Nucleus, cytoplasm, cAMP-dependent Protein Kinase

HANDI Nucleus

As all the four proteins are identified in cellular region: nucleus therefore we can assume they can interact with each other.

Gene Ontology Annotation Similarity:

GLI1 : GLI2 = 0.33

GLI1:KAPCA = 0.25

GLI2:KAPCA = 0.25

Nearly 75% of the interactions in our network were identified to have a common cellular region, hence these proteins have a probability of interaction. Therefore we annotated the interactions score to get the node score i.e. Gene Ontology Annotation Similarity Score:

Protein Gene Ontology Annotation Similarity Score

GLI1 (0.33+0.25)/ 2 = 0.29

GLI2 (0.33+0.25)/2 = 0.29

KAPCA (0.25+ 0.25) /2 = 0.25

**Protein-Protein Interaction Algorithm**

In the sub-network of KAPCA-GLI1-GLI2, structural data is available for GLI1 and KAPCA

Using the protein-protein interaction algorithm the interaction between GLI1:KAPCA = 43.66

Total Network Strength of figure 2: 215 proteins were associated with their structure and the total network strength = 50.6

Therefore the node strength of GLI1 = (43.66)/2 / 50.6 = 0.44
