# Supplementary material for: A systems biology approach to the global analysis of transcription factors in colorectal cancer
Source: BMC Cancer. 2012 Aug 1;12:331. doi: 10.1186/1471-2407-12-331 (PMC3539921; doi:10.1186/1471-2407-12-331)
Supplement: Additional file 2 — Hypergeometric distribution. [file 1471-2407-12-331-S2.docx]

**Additional File II**


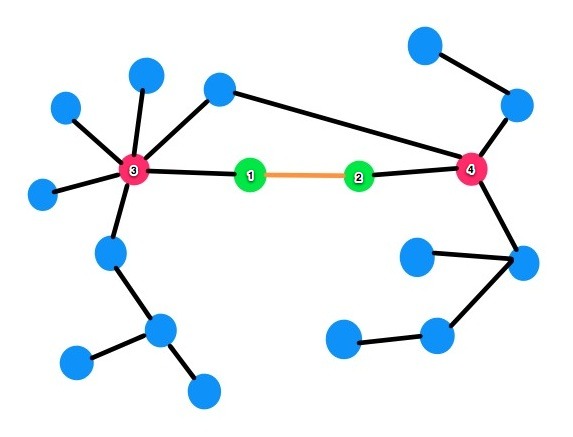


Understanding the hypergeometric associations from the above figure: (i) Consider the node pair (1) and (2). (ii) In the above network these nodes have less degree as compare to nodes 3 and 4. Therefore using degree as the toplogical feature these nodes will not be ranked high. (iii) Node (1) is connected to node (3) which has a higher degree. (iv) Node (2) is connected to node (4) of higher degree. (v) Node (3) and Node (4) share 4 common nodes. Also they are connected to different nodes. (vi) hypergeometric distribution captures this association when computing the strength of Node (1) - Node (2) and ranks this association higher.
