# Supplementary material for: A systems biology approach to the global analysis of transcription factors in colorectal cancer
Source: BMC Cancer. 2012 Aug 1;12:331. doi: 10.1186/1471-2407-12-331 (PMC3539921; doi:10.1186/1471-2407-12-331)
Supplement: Additional file 3 — Few transcription factors and their associated Gene Ontology molecular functions. [file 1471-2407-12-331-S3.docx]

**Additional File III**

**Few transcription factors and their associated Gene Ontology molecular functions**

**_____________________________________________________________**

Gene Symbol GO (Molecular Function)

____________________________________________________________

*CDX2* GO:0003700 [Name: transcription factor activity], GO:0030528 [Name: transcription regulator activity], GO:0043565 [Name: sequence- specific DNA binding], GO:0005515 [Name: protein binding], GO:0003714 [Name: transcription corepressor activity], GO:0003677 [Name: DNA binding]

*CREM*  GO:0003700 [Name: transcription factor activity], GO:0008140 [Name: cAMP response element binding protein binding], GO:0043565 [Name: sequence- specific DNA binding], GO:0046983 [Name: protein dimerization activity], GO:0005515 [Name: protein binding], GO:0003677 [Name: DNA binding]

*DP1* GO:0008408 [Name: 3'-5' exonuclease activity], GO:0003677 [Name: DNA binding], GO:0003887 [Name: DNA-directed DNA polymerase activity],GO:0003700 [Name: transcription factor activity]

*E2F1* GO:0003700 [Name: transcription factor activity],GO:0008134 [Name: transcription factor binding], GO:0016563 [Name: transcription activator activity], GO:0043565 [Name: sequence-specific DNA binding], GO:0005515 [Name: protein binding], GO:0003677 [Name: DNA binding], GO:0003714 [Name: transcription corepressor activity]

*ESR1*  GO:0003700 [Name: transcription factor activity], GO:0005496 [Name: steroid binding], GO:0008270 [Name: zinc ion binding], GO:0030528 [Name: transcription regulator activity], GO:0034056 [Name: estrogen response element binding], GO:0043565 [Name: sequence- specific DNA binding], GO:0003707 [Name: steroid hormone receptor activity], GO:0046872 [Name: metal ion binding], GO:0008134 [Name: transcription factor binding], GO:0030235 [Name: nitric-oxide synthase regulator activity], GO:0032403 [Name: protein complex binding], GO:0005515 [Name: protein binding],GO:0008013 [Name: beta-catenin binding], GO:0030284 [Name: estrogen receptor activity], GO:0042562 [Name: hormone binding], GO:0003677 [Name: DNA binding]

*ESR2* GO:0005496 [Name: steroid binding],GO:0046872 [Name: metal ion binding], GO:0043565 [Name: sequence-specific DNA binding], GO:0005515 [Name: protein binding], GO:0003700 [Name: transcription factor activity], GO:0003707 [Name: steroid hormone receptor activity], GO:0008270 [Name: zinc ion binding],GO:0003677 [Name: DNA binding],GO:0003713 [Name: transcription coactivator activity],GO:0004879 [Name: ligand-dependent nuclear receptor activity],GO:0030284 [Name: estrogen receptor activity],GO:0042562 [Name: hormone binding], ,GO:0048019 [Name: receptor antagonist activity], GO:0003677 [Name: DNA binding]

*ETS1* GO:0003700 [Name: transcription factor activity],GO:0003702 [Name: RNA polymerase II transcription factor activity], GO:0005515 [Name: protein binding], GO:0008134 [Name: transcription factor binding], GO:0043565 [Name: sequence-specific DNA binding]

*ETV3*  GO:0005515 [Name: protein binding], GO:0003674 [Name: molecular_function], GO:0003700 [Name: transcription factor activity], GO:0016564 [Name: transcription repressor activity], GO:0043565 [Name: sequence-specific DNA binding]

*FOXP3*  GO:0003704 [Name: specific RNA polymerase II transcription factor activity], "GO:0003705 [Name: RNA polymerase II transcription factor activity, enhancer binding]", GO:0016564 [Name: transcription repressor activity], GO:0042803 [Name: protein homodimerization activity], GO:0043565 [Name: sequence-specific DNA binding], GO:0051525 [Name: NFAT protein binding], GO:0003700 [Name: transcription factor activity], GO:0003714 [Name: transcription corepressor activity], GO:0008270 [Name: zinc ion binding],GO:0016563 [Name: transcription activator activity], GO:0016566 [Name: specific transcriptional repressor activity], GO:0005515 [Name: protein binding],GO:0003682 [Name: chromatin binding], GO:0003690 [Name: double-stranded DNA binding], GO:0008301 [Name: DNA bending activity], GO:0010843 [Name: promoter binding], GO:0035035 [Name: histone acetyltransferase binding], GO:0051059 [Name: NF-kappaB binding], GO:0046872 [Name: metal ion binding], GO:0042826 [Name: histone deacetylase binding], GO:0046982 [Name: protein heterodimerization activity]

*GATA-1* GO:0003700 [Name: transcription factor activity], GO:0043565 [Name: sequence- specific DNA binding], GO:0008270 [Name: zinc ion binding]

*HAND1* GO:0003677 [Name: DNA binding], GO:0030528 [Name: transcription regulator activity], GO:0003713 [Name: transcription coactivator activity], GO:0010843 [Name: promoter binding], GO:0016564 [Name: transcription repressor activity], GO:0019899 [Name: enzyme binding], GO:0042803 [Name: protein homodimerization activity], GO:0043425 [Name: bHLH transcription factor binding], GO:0003700 [Name: transcription factor activity],GO:0008134 [Name: transcription factor binding]GO:0005515 [Name: protein binding], GO:0042802 [Name: identical protein binding]

*HES1* GO:0005543 [Name: phospholipid binding], GO:0008142 [Name: oxysterol binding], GO:0035091 [Name: phosphoinositide binding], GO:0003677 [Name: DNA binding], GO:0003700 [Name: transcription factor activity], GO:0016564 [Name: transcription repressor activity], GO:0030528 [Name: transcription regulator activity]

*HIF1A*  GO:0005515 [Name: protein binding], GO:0003677 [Name: DNA binding], GO:0003700 [Name: transcription factor activity], GO:0004871 [Name: signal transducer activity], GO:0035035 [Name: histone acetyltransferase binding], GO:0030528 [Name: transcription regulator activity], GO:0042826 [Name: histone deacetylase binding], GO:0008134 [Name: transcription factor binding], GO:0051879 [Name: Hsp90 protein binding], GO:0043565 [Name: sequence- specific DNA binding], GO:0046982 [Name: protein heterodimerization activity], "GO:0003705 [Name: RNA polymerase II transcription factor activity, enhancer binding]"

*IRF1* GO:0003700 [Name: transcription factor activity],GO:0043565 [Name: sequence- specific DNA binding], GO:0003677 [Name: DNA binding]

*IRF7* GO:0003700 [Name: transcription factor activity], GO:0003677 [Name: DNA binding], GO:0003704 [Name: specific RNA polymerase II transcription factor activity], GO:0005515 [Name: protein binding]

*MEF2* GO:0000166 [Name: nucleotide binding], GO:0003924 [Name: GTPase activity], GO:0003746 [Name: translation elongation factor activity], GO:0005525 [Name: GTP binding, GO:0003700 [Name: transcription factor activity], GO:0019901 [Name: protein kinase binding],GO:0043565 [Name: sequence-specific DNA binding], GO:0005515 [Name: protein binding], GO:0016563 [Name: transcription activator activity]

*MEF2C* GO:0005515 [Name: protein binding], GO:0003700 [Name: transcription factor activity], GO:0043565 [Name: sequence-specific DNA binding], GO:0016563 [Name: transcription activator activity], GO:0042826 [Name: histone deacetylase binding], GO:0003702 [Name: RNA polymerase II transcription factor activity]

*MEF2D* GO:0005515 [Name: protein binding],GO:0003700 [Name: transcription factor activity], GO:0043565 [Name: sequence-specific DNA binding], GO:0016563 [Name: transcription activator activity]

*MYOG* GO:0003700 [Name: transcription factor activity], GO:0003677 [Name: DNA binding], GO:0016563 [Name: transcription activator activity], GO:0030528 [Name: transcription regulator activity]

*NANOG* GO:0003700 [Name: transcription factor activity], GO:0043565 [Name: sequence- specific DNA binding], GO:0003677 [Name: DNA binding], GO:0030528 [Name: transcription regulator activity], GO:0005515 [Name: protein binding]

*RUNX3* GO:0005515 [Name: protein binding], GO:0003700 [Name: transcription factor activity], GO:0005524 [Name: ATP binding], GO:0003677 [Name: DNA binding]

*SMAD2* GO:0005515 [Name: protein binding],"GO:0030618 [Name: transforming growth factor beta receptor, pathway-specific cytoplasmic mediator activity]", GO:0005160 [Name: transforming growth factor beta receptor binding], GO:0031625 [Name: ubiquitin protein ligase binding], GO:0042803 [Name: protein homodimerization activity], GO:0070412 [Name: R-SMAD binding], GO:0003690 [Name: double- stranded DNA binding], GO:0003700 [Name: transcription factor activity], GO:0005518 [Name: collagen binding], GO:0008134 [Name: transcription factor binding], GO:0010843 [Name: promoter binding], GO:0016563 [Name: transcription activator activity], GO:0019901 [Name: protein kinase binding], GO:0070410 [Name: co-SMAD binding], GO:0003677 [Name: DNA binding], GO:0003682 [Name: chromatin binding], GO:0034713 [Name: type I transforming growth factor beta receptor binding], GO:0070411 [Name: I-SMAD binding], GO:0046332 [Name: SMAD binding]

*SMAD3*  GO:0003690 [Name: double-stranded DNA binding], GO:0003700 [Name: transcription factor activity], "GO:0030618 [Name: transforming growth factor beta receptor, pathway-specific cytoplasmic mediator activity]", GO:0008134 [Name: transcription factor binding], GO:0016563 [Name: transcription activator activity], GO:0019901 [Name: protein kinase binding], GO:0042803 [Name: protein homodimerization activity], GO:0070410 [Name: co-SMAD binding], GO:0005160 [Name: transforming growth factor beta receptor binding], GO:0005518 [Name: collagen binding], GO:0010843 [Name: promoter binding], GO:0031625 [Name: ubiquitin protein ligase binding], GO:0070412 [Name: R-SMAD binding], GO:0005515 [Name: protein binding], GO:0003706 [Name: ligand-regulated transcription factor activity], GO:0043565 [Name: sequence-specific DNA binding], GO:0008013 [Name: beta-catenin binding], GO:0016566 [Name: specific transcriptional repressor activity]

*SMAD4* GO:0005515 [Name: protein binding], GO:0003700 [Name: transcription factor activity], "GO:0030616 [Name: transforming growth factor beta receptor, common-partner cytoplasmic mediator activity]", GO:0005518 [Name: collagen binding], GO:0042802 [Name: identical protein binding], GO:0010843 [Name: promoter binding], GO:0016563 [Name: transcription activator activity], GO:0070411 [Name: I-SMAD binding], GO:0003677 [Name: DNA binding], GO:0003682 [Name: chromatin binding], GO:0042803 [Name: protein homodimerization activity], GO:0043565 [Name: sequence-specific DNA binding], GO:0070412 [Name: R-SMAD binding], GO:0005515 [Name: protein binding]

*SMAD7* GO:0005518 [Name: collagen binding], GO:0008013 [Name: beta-catenin binding], GO:0010843 [Name: promoter binding], "GO:0030617 [Name: transforming growth factor beta receptor, inhibitory cytoplasmic mediator activity]", GO:0031625 [Name: ubiquitin protein ligase binding], GO:0034713 [Name: type I transforming growth factor beta receptor binding], GO:0048185 [Name: activin binding], GO:0070411 [Name: I-SMAD binding]

*SP1* GO:0003676 [Name: nucleic acid binding] , GO:0008270 [Name: zinc ion binding], GO:0005515 [Name: protein binding], GO:0003700 [Name: transcription factor activity], GO:0008022 [Name: protein C- terminus binding], GO:0042802 [Name: identical protein binding], GO:0010843 [Name: promoter binding], GO:0035035 [Name: histone acetyltransferase binding], GO:0043565 [Name: sequence-specific DNA binding], GO:0003677 [Name: DNA binding], GO:0003690 [Name: double- stranded DNA binding], GO:0003702 [Name: RNA polymerase II transcription factor activity], GO:0046872 [Name: metal ion binding], GO:0016563 [Name: transcription activator activity], GO:0035326 [Name: enhancer binding], GO:0042803 [Name: protein homodimerization activity], GO:0042826 [Name: histone deacetylase binding]

*SP3* GO:0005515 [Name: protein binding], GO:0046872 [Name: metal ion binding], GO:0003677 [Name: DNA binding], GO:0003682 [Name: chromatin binding], GO:0003690 [Name: double-stranded DNA binding], GO:0008270 [Name: zinc ion binding], GO:0016564 [Name: transcription repressor activity], GO:0016563 [Name: transcription activator activity] , GO:0003676 [Name: nucleic acid binding]

*SRF* GO:0005515 [Name: protein binding], GO:0043565 [Name: sequence- specific DNA binding], GO:0003700 [Name: transcription factor activity], GO:0003704 [Name: specific RNA polymerase II transcription factor activity], GO:0008134 [Name: transcription factor binding], GO:0010736 [Name: serum response element binding], GO:0042803 [Name: protein homodimerization activity]

*STAT1* GO:0003677 [Name: DNA binding], GO:0004871 [Name: signal transducer activity], GO:0043565 [Name: sequence-specific DNA binding], GO:0005515 [Name: protein binding], GO:0005509 [Name: calcium ion binding], GO:0003700 [Name: transcription factor activity]

*STAT3* GO:0005509 [Name: calcium ion binding], GO:0003677 [Name: DNA binding], GO:0046983 [Name: protein dimerization activity], GO:0003700 [Name: transcription factor activity], GO:0004871 [Name: signal transducer activity], GO:0016563 [Name: transcription activator activity], GO:0019901 [Name: protein kinase binding], GO:0008134 [Name: transcription factor binding], GO:0005515 [Name: protein binding], GO:0043565 [Name: sequence-specific DNA binding]

*STAT4* GO:0005515 [Name: protein binding], GO:0005509 [Name: calcium ion binding], GO:0004871 [Name: signal transducer activity], GO:0003700 [Name: transcription factor activity], GO:0043565 [Name: sequence- specific DNA binding]

*STAT5A* GO:0005509 [Name: calcium ion binding], GO:0004871 [Name: signal transducer activity], GO:0005515 [Name: protein binding], GO:0003677 [Name: DNA binding], GO:0003700 [Name: transcription factor activity]

*STAT6*  GO:0005515 [Name: protein binding], GO:0003700 [Name: transcription factor activity], GO:0043565 [Name: sequence-specific DNA binding],GO:0005509 [Name: calcium ion binding], GO:0004871 [Name: signal transducer activity]

*TBP* GO:0010843 [Name: promoter binding], GO:0016251 [Name: general RNA polymerase II transcription factor activity], GO:0003677 [Name: DNA binding], GO:0008134 [Name: transcription factor binding], GO:0003700 [Name: transcription factor activity], GO:0003702 [Name: RNA polymerase II transcription factor activity], GO:0005515 [Name: protein binding], VDR GO:0003707 [Name: steroid hormone receptor activity]

*VDR* GO:0046872 [Name: metal ion binding], GO:0008434 [Name: vitamin D3 receptor activity], GO:0030528 [Name: transcription regulator activity], GO:0070644 [Name: vitamin D response element binding], GO:0005515 [Name: protein binding], GO:0003677 [Name: DNA binding], GO:0003700 [Name: transcription factor activity], GO:0008270 [Name: zinc ion binding], GO:0043565 [Name: sequence- specific DNA binding], GO:0046965 [Name: retinoid X receptor binding]

*XBP1* GO:0043565 [Name: sequence-specific DNA binding], GO:0003677 [Name: DNA binding], GO:0003700 [Name: transcription factor activity], GO:0046983 [Name: protein dimerization activity]

*p53* GO:0003677 [Name: DNA binding], GO:0003700 [Name: transcription factor activity], GO:0003702 [Name: RNA polymerase II transcription factor activity], GO:0005515 [Name: protein binding]

________________________________________________________________________
