# Supplementary material for: A systems biology approach to the global analysis of transcription factors in colorectal cancer
Source: BMC Cancer. 2012 Aug 1;12:331. doi: 10.1186/1471-2407-12-331 (PMC3539921; doi:10.1186/1471-2407-12-331)
Supplement: Additional file 4 — Nodes with highest number of connections identified for each functional group (defined by MetaCoreTMin GeneGO). [file 1471-2407-12-331-S4.docx]

**Additional File IV**

**Nodes with highest number of connections identified for each functional group (defined by MetaCore^TM^ in GeneGO)**

**Functional group Transcription Factors Connectivity**

Apoptosis and Survival *c-Jun* 12

*NF-kB* 11

*p53* 11

*E2F1*  9

Cell Adhesion *c-Myc* 2

Cell Cycle *E2F1* 9

*SP1* 7

*ESR1(nuclear)* 6

*E2F1/DP1 complex* 6

Cytoskeleton Remodeling *GATA-1* 5

*c-Jun* 3

*SMAD3* 3

Development *STAT3* 39

*c-Jun* 30

*Elk-1* 30

*NF-kB*  20

*ESR1(nuclear)*  19

*c-Myc* 17

DNA damage *p53* 26

G-protein signaling *c-Jun* 9

Immune-Response *STAT1* 32

*STAT3* 26

*NF-kB* 20

Signal Transduction *SMAD2* 7

*SMAD4* 6

Transcription *HIF-1A* 6

*PPAR-alpha* 4

Other Functional Group *SMAD3* 13

*STAT3* 11

*MEF2D* 8
