# Supplementary material for: A systems biology approach to the global analysis of transcription factors in colorectal cancer
Source: BMC Cancer. 2012 Aug 1;12:331. doi: 10.1186/1471-2407-12-331 (PMC3539921; doi:10.1186/1471-2407-12-331)
Supplement: Additional file 5 — Functional group transcription factor distribution. Transcription factors are arranged in decreasing order with respect to their connectivity in each functional group. [file 1471-2407-12-331-S5.docx]

**Additional File V**

**Functional group transcription factor distribution**

**(Transcription factors are arranged in decreasing order with respect to their connectivity in each functional group)**

Functional groups (Number of TFs) TFs

Apoptosis and Survival (20) *c-Jun, NF-kB, p53, E2F1, NF-kB p50/p65, NF-kBp52/ReIB, NF-kB1, ReIB, C/EBPzeta, NF-kB1 (p50/p65), p73, NF-kB(p50/p50), NF-kB(p50/p51), NF-kB1(p50), XBP1, AFX1, c-Jun/c-Fos, c-Myc, Elk-1, ESR1(nuclear), FKHR, NF-kB1(p50)*

Cell Adhesion (7) *NF-kB, c-Myc, c-Jun, STAT1, Elk-1,*

*CDX2, SP1*

Cell Cycle (15) *E2F1, SP1, E2F1/DP1complex,*

*ESR1(nuclear), c-Jun, SMAD3, DP1, c-Myc, p53, ATF-2/c-Jun, c-Jun/c-Fos, SMAD2, SMAD4, E2F4/ DP1 complex, Elk-1*

Chemotaxis (2) *Elk-1, STAT3*

Cytoskeleton Remodeling (10) *GATA-1, c-Jun, SMAD3, c-Myc*

*SMAD2, Tcf(Lef), Elk-1, FOXO3A, p53, SP1*

Development ( 54) *STAT3, c-Jun, Elk-1, NF-kB,*

*ESR1 (nuclear), c-Myc, STAT1, MEF2, SMAD3, GCR-alpha, SMAD2, c-Jun/c-Fos, ATF-2, Tcf(Lef), SP1, FOXO3A, SMAD4, MEF2A, ESR2, GLI-1, IRF1, MEF2D, MYOG, SRF, ATF-1, FKHR, GATA-4, GL1-2, NF-kB(p50/p65), NF-kB(p52/ReIB), PU.1, VDR, AML1(RUNX1), AP-1, C/EBPzeta, c-Fos, c-Myb, E2F1, EPAS1/ ARNT, GATA-1, HAND1, HES1, HIF1A, Lef-1, MAD, MYOD, NANOG, NRSF,p53, PPAR-beta, SLUG, SP3, STAT5A, STAT6*

DNA Damage (9) *p53, c-Myc, ESR1, SP1, STAT1, ATF-1,*

*E2F1, NF-kB, SP3*

G-protein-signaling (8) *ATF-2, c-Jun, ESR1, c-Jun/c-Fos, Elk-1, NF-kB, MEF2C, STAT3*

Immune Response (48) *STAT1, c-Jun, STAT3, NF-kB, c-Jun/c-Fos, AP-1, Elk-1, MEF2, STAT4, ATF-2, CREM(repressors), c-Myc, IRF1, NF-kB(p50/p65), MEF2A, p53, MEF2C, ETS1, ETV3, GCR-alpha, STAT6, c-Myb, CREM(activators), MEF2D, ATF-2/c-Jun, CBP, c-Maf, CREM, CRP2, ERM, FasR(CD95), FKHR, FosB, FOXO3A, GATA-3, IRF7, ISGF3, NF-kB(p50/p50), NF-kB1(p50), NUR77, RUNX3, SMAD3, SMAD4, SMAD7, SP1, SP3, SRF, STAT5*

Muscle Contraction (1) *NF-kB*

Signal Transduction (11) *SMAD2, SMAD4, p53, PTEN, FOXO3A, MEF2, NF-kB, Elk-1, c-Jun, NUR77*

Transcription ( 24) *HIF-1A, PPAR-alpha, c-Jun/c-Fos, SP1,*

*AP-1, c-Jun, NF-kB, NRF2, TBP, TFIID, AP-2A, c-Myc, GCR-alpha, HIF1A, MeF-2, MYOD, p53, PPAR- alpha/RXR-alpha, PPAR-beta(delta), PPAR-beta(delta)/RXR-alpha, RAR- alpha/RXR-alpha, RAR-alpha/RXR- beta, RAR-alpha/TR-alpha, RAR- beta/RXR-alpha*

Other Functional Groups (31) *AP-1, c-Jun, c-Jun/c-Fos, Elk-1,*

*FOXP3, FXR/RXR-alpha, GATA-4, HAND1, HNF1-alpha, HNF3,*

*HNF3-alpha, HNF3-beta, HNF3-gamma, HNF4-alpha, IRF1,*

*MEF2A, MEF2C, MEF2D, NF-kBp50/p65, p53, RARalpha,*

*RARbeta, RAR-beta/RXR-alpha, SHP, SMAD2, SMAD3, SMAD4,*

*SP1, STAT1, STAT3, VDR/RXR-alpha,*
