# Supplementary material for: A systems biology approach to the global analysis of transcription factors in colorectal cancer
Source: BMC Cancer. 2012 Aug 1;12:331. doi: 10.1186/1471-2407-12-331 (PMC3539921; doi:10.1186/1471-2407-12-331)
Supplement: Additional file 6 — Analysis of transcription factors identified with prognostic value in CRC. [file 1471-2407-12-331-S6.docx]

**Additional File VI**

**Analysis of transcription factors identified with prognostic value in CRC**

# Transcription Prognostic Value Identified Reference

**Factors** **CRC**

*SP1* Studies in Progress

*Elk-1* Might [[113](#_ENREF_113)]

*NF-kB* Yes [[114](#_ENREF_114)]

*p53* Yes [[115](#_ENREF_115)]

*c-Myc* Yes [[115](#_ENREF_115)]

*SMAD4* Yes [[116](#_ENREF_116)]

*STAT3* Poor Prognosis [[117](#_ENREF_117)]

STAT1 Y (recent) [[118](#_ENREF_118)]

*HIF1A*  Y [[119](#_ENREF_119)]

*NANOG* Poor Prognosis [[120](#_ENREF_120)]

*SLUG* Poor Prognosis [[121](#_ENREF_121)]

Studies are being carried out and some others being tested

*RUNX1, Lef-1, GLI-1, PU.1, MYOD, FOXO3, E2F1, ATF-2, IRF1, SP3, MEF2, ESR1, AP-1*
